# Integrated Blood Inflammatory Ratios and Cerebrospinal Fluid Blood‒Brain Barrier Dysfunction Predict Relapse Risk in Neuromyelitis Optica Spectrum Disorder

**DOI:** 10.1002/brb3.71481

**Published:** 2026-06-12

**Authors:** Xingyue Zheng, Jing Shi, Hao Yin, Huiqun Song, Quanhua Zhai, Cuiping Si, Jianwen Chen, Peixue Li, Lei Zhang, Yuzhi Li, Chunbo Dong, Huakun Liu

**Affiliations:** ^1^ Department of Neurology, The First Affiliated Hospital of Dalian Medical University Dalian Medical University Dalian China; ^2^ Department of Neurology Jining No. 1 People's Hospital Jining China

**Keywords:** blood–brain barrier dysfunction, Cox regression, neuromyelitis optica spectrum disorder, nomogram, peripheral inflammatory ratios, relapse risk

## Abstract

**Background:**

Relapse is the primary driver of irreversible disability accumulation in neuromyelitis optica spectrum disorder (NMOSD). Although aquaporin‐4 immunoglobulin G (AQP4‐IgG) is central to disease diagnosis and pathogenesis, reliable tools for individualized relapse risk stratification remain limited. Given emerging evidence that blood–brain barrier (BBB) dysfunction and systemic immune activation play key and interconnected roles in NMOSD pathophysiology, we aimed to develop and internally validate an integrated prognostic model incorporating these dimensions to predict relapse risk.

**Methods:**

In this retrospective cohort study, 152 patients with NMOSD were enrolled and followed longitudinally for time to first relapse. Baseline peripheral inflammatory indices, including the neutrophil‐to‐lymphocyte ratio (NLR) and monocyte‐to‐lymphocyte ratio (MLR), as well as cerebrospinal fluid (CSF) parameters reflecting BBB integrity, were collected at study entry. Independent predictors of time to first relapse were identified using multivariable Cox proportional hazards regression. Three hierarchical prognostic models were constructed and compared: a clinical model, a clinical‐CSF model, and an integrated clinical‐CSF‐blood inflammatory model. Model discrimination was evaluated using time‐dependent receiver operating characteristic (ROC) analysis. Internal validation was performed using 1000 bootstrap resamples, and clinical utility was assessed by decision curve analysis (DCA).

**Results:**

Patients who experienced relapse exhibited more pronounced BBB dysfunction and higher systemic inflammatory activation at baseline. Model discrimination improved with the sequential incorporation of CSF indices and inflammatory markers, with AUCs of 0.693 for the clinical model, 0.744 for the clinical‐CSF model, and 0.850 for the integrated model. The final nomogram demonstrated good discrimination (bootstrap‐corrected *C*‐index 0.811), good calibration, and favorable clinical utility. AQP4‐IgG seropositive patients had a higher relapse risk; importantly, the integrated model retained predictive performance in this subgroup, indicating added prognostic value beyond serological status. Exploratory analyses further suggested that higher AQP4‐IgG titers were associated with increased relapse risk.

**Conclusions:**

An integrated nomogram incorporating peripheral inflammatory ratios and BBB‐related CSF indices enables individualized relapse risk prediction in NMOSD. The model provides incremental prognostic value beyond AQP4‐IgG serostatus and may support risk‐adapted clinical management.

## Graphical abstract

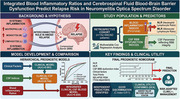

**Abbreviations**
AQP4: aquaporin‐4
AQP4‐IgG: aquaporin‐4 immunoglobulin G
BBB: blood–brain barrier
BioT: biologic immunotherapy
CBA: cell‐based assay
CI: confidence interval
CIT: conventional immunosuppressive therapy
CRP: C‐reactive protein
CSF: cerebrospinal fluid
CSF IgG‐24h: 24‐h intrathecal immunoglobulin G synthesis rate
DCA: decision curve analysis
EDSS: Expanded Disability Status Scale
HR: hazard ratio
IVIG: intravenous immunoglobulin
MLL: multifocal lesion location
MLR: monocyte‐to‐lymphocyte ratio
NLR: neutrophil‐to‐lymphocyte ratio
NMOSD: neuromyelitis optica spectrum disorder
NoMT: no maintenance therapy
ONL: optic neuritis–predominant lesion
PLEX: plasma exchange
PLR: platelet‐to‐lymphocyte ratio
QAlb: cerebrospinal fluid/serum albumin quotient
ROC: receiver operating characteristic
SCL: spinal cord lesion

## 1 Introduction

Neuromyelitis optica spectrum disorder (NMOSD) is a severe autoimmune astrocytopathy of the central nervous system characterized by recurrent attacks of optic neuritis and myelitis, leading to substantial and often irreversible neurological disability (Wingerchuk et al. 2015). Unlike progressive neurodegenerative diseases, long‐term disability accumulation in NMOSD is predominantly attack‐related, making relapse prevention a central therapeutic objective (Mealy et al. 2014). Accurate identification of patients at high risk of relapse therefore represents a critical unmet need in NMOSD management.

Aquaporin‐4 immunoglobulin G (AQP4‐IgG) plays a pivotal pathogenic role in NMOSD by targeting astrocytic end‐feet and initiating complement‐dependent cytotoxicity once it gains access to the central nervous system (Lennon et al. 2005; Papadopoulos and Verkman 2012). Although AQP4‐IgG seropositivity is indispensable for diagnosis and disease classification, its value for individualized relapse prediction remains limited. Considerable heterogeneity exists among seropositive patients in relapse frequency and treatment response, indicating that antibody status alone does not adequately capture relapse susceptibility (Sato et al. 2014; Wei et al. 2024). This heterogeneity underscores the need for additional biomarkers that reflect the biological conditions permitting antibody‐mediated central nervous system injury.

Peripheral systemic inflammation has emerged as an important contributor to disease activity in autoimmune neuroinflammatory disorders. Composite inflammatory ratios derived from routine blood tests, such as the neutrophil‐to‐lymphocyte ratio (NLR) and monocyte‐to‐lymphocyte ratio (MLR), reflect a shift toward innate immune activation and have been associated with disease activity, relapse risk, and disability progression across multiple autoimmune conditions (Xie et al. 2021; Cabanillas‐Lazo et al. 2023; Wu et al. 2025; Gasparyan et al. 2011). In NMOSD, growing evidence suggests that elevated NLR and MLR are linked to higher relapse rates and worse neurological outcomes, highlighting their potential utility as accessible prognostic biomarkers (Wu et al. 2025; Zhang et al. 2024; Miyamoto et al. 2025). However, peripheral inflammatory markers alone do not directly reflect central nervous system vulnerability to antibody‐mediated injury.

The integrity of the blood–brain barrier (BBB) is a critical determinant of whether circulating pathogenic antibodies and immune cells can access the central nervous system. The cerebrospinal fluid/serum albumin quotient (QAlb) is a widely accepted surrogate marker of BBB permeability and has been shown to reflect barrier dysfunction in inflammatory neurological diseases (Reiber and Peter 2001; Johnsson et al. 2025; Liang et al. 2020). In NMOSD, increased QAlb has been associated with disease severity, cerebrospinal fluid (CSF) inflammatory abnormalities, and poor clinical outcomes, supporting the concept that BBB vulnerability may represent a permissive state for disease reactivation (Johnsson et al. 2025; Tanaka et al. 2020; Shimizu and Nakamori 2024; Sun et al. 2025; Takata et al. 2021; Rodin and Chitnis 2024). Importantly, BBB dysfunction may interact with systemic inflammatory activation, forming a pathological interface through which peripheral immune processes amplify central neuroinflammation (Sun et al. 2025; Winkler et al. 2021; Liu et al. 2023).

Despite increasing recognition of the roles of systemic inflammation and BBB dysfunction in NMOSD, these biologically complementary domains have largely been investigated in isolation. Few studies have integrated peripheral inflammatory markers and CSF indices within a unified prognostic framework, and their combined value for individualized relapse risk prediction remains insufficiently explored (Li et al. 2025). Given the multifactorial nature of relapse pathogenesis, integrating markers of systemic immune activation and central barrier integrity may provide a more comprehensive representation of relapse susceptibility.

Accordingly, the present study aimed to investigate the associations of blood inflammatory ratios and CSF BBB dysfunction with subsequent relapse in patients with NMOSD. We further sought to develop and internally validate a multivariable Cox regression‐based prognostic model integrating clinical characteristics, CSF indices, and peripheral inflammatory markers. By constructing a nomogram and evaluating its discrimination, calibration, and clinical utility, we aimed to provide an individualized and clinically applicable tool for relapse risk stratification in NMOSD.

## 2 Methods

### 2.1 Study Design and Participants

This retrospective cohort study consecutively enrolled patients diagnosed with NMOSD in a real‐world clinical setting at Jining No. 1 People's Hospital, a tertiary referral center in China, between January 2017 and December 2023. All patients fulfilled the 2015 International Panel for NMO Diagnosis (IPND) criteria (Wingerchuk et al. 2015). Patients were eligible if they met the following criteria: (1) complete baseline clinical, laboratory, and CSF data obtained during the index hospitalization and (2) at least 18 months of follow‐up or a documented relapse during follow‐up. Patients with concomitant systemic autoimmune diseases, active infections at baseline, malignancy, or incomplete follow‐up data were excluded.

A total of 212 patients were assessed for eligibility, and 63 were excluded based on predefined criteria: 23 patients were MOG‐IgG‐positive, 3 were GFAP‐IgG‐positive, 12 were seronegative with other diagnoses, 9 had insufficient CSF data, 7 were lost to follow‐up, and 9 had inadequate clinical records. Ultimately, 152 patients were enrolled and followed for 18 months. During follow‐up, 68 patients experienced a first relapse, while 84 patients remained relapse‐free. A detailed flowchart illustrating patient screening, exclusion criteria, enrollment, and follow‐up is provided in Figure S1.

The study was approved by the institutional ethics committee of Jining No. 1 People's Hospital (Approval no.: KYLL‐202307‐102), and the requirement for written informed consent was waived due to the retrospective design.

### 2.2 Clinical Data Collection

Baseline demographic and clinical variables were extracted from electronic medical records, including age, sex, disease duration, baseline Expanded Disability Status Scale (EDSS) score, AQP4‐IgG serostatus, and disease phenotype. Lesion distribution at attack onset was categorized based on clinical presentation and neuroimaging findings into optic neuritis–predominant lesions (ONLs), spinal cord lesions (SCL), or multifocal lesions involving two or more central nervous system regions (multifocal lesion location [MLL]).

Neurological disability was assessed using the EDSS. For descriptive and stratified analyses, patients were categorized into a mild disability group (EDSS < 4) and a moderate‐to‐severe disability group (EDSS ≥ 4), reflecting clinically meaningful functional impairment.

### 2.3 Acute Attack Management and Maintenance Therapy

Acute attacks were managed according to contemporary NMOSD treatment guidelines. All patients received high‐dose intravenous methylprednisolone (1 g/day for 3–5 days), followed by oral corticosteroid tapering. Plasma exchange (PLEX; five to seven sessions) was administered in patients with inadequate response to corticosteroids or severe attacks. Intravenous immunoglobulin (IVIG) was used as rescue therapy at the discretion of the treating physician.

Post‐discharge maintenance therapy was categorized into three groups: (1) no standardized immunosuppressive treatment, (2) conventional immunosuppressive therapy (CIT) (e.g., azathioprine, mycophenolate mofetil, or oral corticosteroids), and (3) biologic immunotherapy (e.g., rituximab or other targeted agents). The maintenance treatment category was included as a covariate in survival analyses to partially account for treatment‐related confounding.

### 2.4 Laboratory Assessments

#### 2.4.1 Blood Inflammatory Markers

Peripheral blood samples were collected at baseline during index hospitalization before initiation of immunomodulatory therapy. Absolute neutrophil, lymphocyte, monocyte, and platelet counts were obtained from routine complete blood counts. Blood inflammatory ratios were calculated as follows: NLR, MLR and platelet‐to‐lymphocyte ratio (PLR). Serum C‐reactive protein (CRP) levels were also recorded.

#### 2.4.2 AQP4‐IgG Detection

AQP4‐IgG was detected using a standardized cell‐based assay (CBA). Patients were classified as AQP4‐IgG positive or negative according to assay results. AQP4‐IgG serostatus was considered a core disease‐defining and prognostic variable.

#### 2.4.3 CSF Analysis and BBB Assessment

CSF samples were obtained during the acute phase before initiation of immunotherapy. CSF and paired serum albumin concentrations were measured using standard laboratory methods. BBB integrity was assessed using the QAlb, calculated as:

$$\mathrm{QAlb}=\mathrm{CSF}\;\mathrm{albumin}/\mathrm{serum}\;\mathrm{albumin}\times 10^{-3}$$

Because QAlb physiologically increases with age, the upper limit of the reference range was determined using an age‐adjusted formula:

$$QAlb_{upper}=4+\left(age/15\right)$$

Values exceeding the age‐adjusted upper limit were defined as elevated QAlb, indicating increased BBB permeability, whereas values within the reference range were considered normal, reflecting preserved BBB integrity.

Additional CSF immune indices, including the IgG index and 24‐h intrathecal IgG synthesis rate, were calculated using standard formulas and analyzed as continuous variables in secondary analyses.

#### 2.4.4 Outcome Definition and Follow‐Up

The primary outcome was time to first relapse, defined as the interval from index hospitalization to the occurrence of a new neurological event attributable to NMOSD, as confirmed by treating neurologists, accompanied by objective neurological deterioration and not explained by alternative diagnoses. Patients without relapse were censored at the last follow‐up visit. Follow‐up duration was calculated in months.

### 2.5 Statistical Analysis

Continuous variables were summarized as the mean ± standard deviation or median (interquartile range), as appropriate, and compared using Student's *t*‐test or the Mann–Whitney *U* test. Categorical variables were expressed as counts (percentages) and compared using the *χ* ^2^ test or Fisher's exact test.

Time‐to‐event analyses were performed using Cox proportional hazards regression (Prentice and Zhao 2021). Univariable Cox regression was first conducted to identify candidate predictors of relapse. Variables with *p* < 0.10 in univariable analyses, along with clinically relevant covariates, were entered into multivariable Cox regression models. The proportional hazards assumption was assessed using Schoenfeld residuals (O'Quigley and Flandre 1994).

### 2.6 Additional Analyses According to AQP4‐IgG Serostatus

To address the potential influence of AQP4‐IgG serological status, additional analyses were performed. Kaplan‒Meier survival analysis with log rank testing was used to compare relapse risk between AQP4‐IgG seropositive and seronegative patients.

In patients with available quantitative antibody measurements, exploratory analyses were conducted to evaluate the association between AQP4‐IgG titers and relapse risk using univariable Cox proportional hazards regression. Antibody titers were dichotomized into high and low groups based on the median value.

Subgroup analyses were further conducted in AQP4‐IgG seropositive patients. Specifically, multivariable Cox proportional hazards regression was reperformed in this subgroup using the same covariates and modeling strategy as in the primary analysis to evaluate the stability of predictor effects. Hazard ratios (HRs) with 95% confidence intervals (CIs) were estimated and visualized using forest plots.

Interaction terms between AQP4‐IgG serostatus and key predictors (including QAlb, NLR, and MLR) were incorporated into Cox models to evaluate potential effect modification.

### 2.7 Model Development and Validation

Three hierarchical Cox regression models were constructed: (1) a base clinical model, (2) a clinical plus CSF model, and (3) an integrated clinical‐CSF‐blood inflammatory marker model. Model discrimination was evaluated using Harrell's concordance index (*C*‐index) to quantify the ability of the models to correctly rank patients according to relapse risk (Harrell et al. 1996).

Internal validation was performed using 1000 bootstrap resamples to estimate optimism‐corrected *C*‐index values (Steyerberg and Harrell 2016). Model calibration was assessed using calibration curves comparing predicted and observed relapse probabilities at 12 months (Steyerberg et al. 2010). Time‐dependent receiver operating characteristic (ROC) curves were generated at 6 and 12 months to evaluate predictive performance over time (Heagerty et al. 2000). Decision curve analysis (DCA) was conducted to assess the net clinical benefit of the final model across a range of clinically relevant threshold probabilities (Vickers and Elkin 2006).

A nomogram was developed based on the final multivariable Cox model to facilitate individualized relapse risk prediction in routine clinical practice (Huang et al. 2023). All statistical analyses were performed using R software (version 4.5.2), and a two‐sided *p* < 0.05 was considered statistically significant.

## 3 Results

### 3.1 Baseline Characteristics of Relapsed and Non‐Relapsed Patients

A total of 152 patients with NMOSD were included in the final analysis. During follow‐up, 68 patients (44.7%) experienced at least one relapse, whereas 84 patients (55.3%) remained relapse‐free. Baseline demographic, clinical, laboratory, and treatment‐related characteristics stratified by relapse status are summarized in Table 1.

**TABLE 1:** Baseline demographic, clinical, cerebrospinal fluid, and blood inflammatory characteristics of NMOSD patients stratified by relapse status.

	Relapse status				
Variable	Overall, *N* = 152	Non‐relapsed, *N* = 84 (55%)	Relapsed, *N* = 68 (45%)	Statistics	*p*
Sex				7.19	0.007
Male	37 (24.34%)	28 (33.33%)	9 (13.24%)		
Female	115 (75.66%)	56 (66.67%)	59 (86.76%)		
Age	45.00 [33.00, 59.25]	47.50 [34.75, 61.25]	44.50 [30.75, 58.25]	3174.00	0.239
Location				11.01	0.004
ONL	39 (25.66%)	14 (16.67%)	25 (36.76%)		
SCL	77 (50.66%)	52 (61.90%)	25 (36.76%)		
MLL	36 (23.68%)	18 (21.43%)	18 (26.47%)		
Serum AQP4‐IgG				29.07	< 0.001
Negative	57 (37.50%)	48 (57.14%)	9 (13.24%)		
Positive	95 (62.50%)	36 (42.86%)	59 (86.76%)		
Qalb group				10.50	0.001
Normal	90 (59.21%)	60 (71.43%)	30 (44.12%)		
Elevate	62 (40.79%)	24 (28.57%)	38 (55.88%)		
Qalb	5.63 [3.69, 9.34]	4.74 [3.30, 7.07]	7.46 [4.85, 10.51]	1781.00	< 0.001
QIgG	3.19 [2.08, 4.97]	2.86 [1.93, 3.93]	3.67 [2.25, 5.72]	2208.50	0.016
QIgA	1.66 [1.09, 2.54]	1.61 [1.13, 1.90]	1.94 [1.06, 3.58]	2302.00	0.039
QIgM	0.81 [0.39, 1.68]	0.71 [0.35, 1.41]	0.87 [0.42, 2.64]	2455.50	0.138
CSF‐IgG‐index	0.53 [0.46, 0.64]	0.53 [0.47, 0.71]	0.52 [0.46, 0.58]	3177.00	0.235
CSF‐IgG‐24h	2.24 [0.02, 5.48]	2.09 [‐0.31, 4.62]	2.48 [0.43, 6.38]	2533.50	0.233
NLR	2.87 [1.78, 4.89]	2.03 [1.42, 3.06]	4.71 [3.18, 5.81]	1001.00	< 0.001
MLR	0.29 [0.20, 0.38]	0.21 [0.17, 0.30]	0.33 [0.30, 0.41]	1265.50	< 0.001
PLR	122.37 [92.18, 167.21]	115.04 [90.20, 160.68]	123.75 [95.23, 180.88]	2613.50	0.370
CRP	2.36 [1.76, 3.37]	2.24 [1.63, 3.38]	2.50 [1.98, 3.30]	2530.5	0.198
EDSS group				3.20	0.074
Mild	87 (57.24%)	54 (64.29%)	33 (48.53%)		
Moderate‐severe	65 (42.76%)	30 (35.71%)	35 (51.47%)		
EDSS score	3.50 [3.00, 4.50]	3.00 [2.00, 4.00]	4.00 [3.00, 4.50]	2204.50	0.005
Maintenance therapy				6.37	0.041
NoMT	20 (13.16%)	6 (7.14%)	14 (20.59%)		
CIT	109 (71.71%)	63 (75.00%)	46 (67.65%)		
BioT	23 (15.13%)	15 (17.86%)	8 (11.76%)		
Acute attack therapy					0.449
IVMP	108 (71.05%)	56 (66.67%)	52 (76.47%)		
IVIG	38 (25.00%)	24 (28.57%)	14 (20.59%)		
PLEX	6 (3.95%)	4 (4.76%)	2 (2.94%)		

*Note*: Baseline characteristics of 152 patients with neuromyelitis optica spectrum disorder (NMOSD), stratified according to relapse occurrence during follow‐up. Data are presented as the mean ± standard deviation, median (interquartile range), or number (percentage), as appropriate. Continuous variables were compared using Student's *t*‐test or the Mann–Whitney *U* test, and categorical variables were compared using the *χ* ^2^ test or Fisher's exact test.

Abbreviations: AQP4‐IgG, aquaporin‐4 immunoglobulin G; CRP, C‐reactive protein; CSF, cerebrospinal fluid; EDSS, Expanded Disability Status Scale; IVIG, intravenous immunoglobulin; MLL, multifocal lesions; MLR, monocyte‐to‐lymphocyte ratio; NLR, neutrophil‐to‐lymphocyte ratio; NMOSD, neuromyelitis optica spectrum disorder; ONL, optic neuritis–predominant lesions; PLEX, plasma exchange; PLR, platelet‐to‐lymphocyte ratio; QAlb, cerebrospinal fluid/serum albumin quotient; QIgA, cerebrospinal fluid/serum immunoglobulin A quotient; QIgG, cerebrospinal fluid/serum immunoglobulin G quotient; QIgM, cerebrospinal fluid/serum immunoglobulin M quotient; SCL, spinal cord lesions.

Female sex was more frequent among patients who relapsed than among those who remained relapse‐free (86.8% vs. 66.7%, *p* = 0.007). Lesion distribution differed significantly between groups (*p* = 0.004), with ONLs being more common in relapsed patients, whereas isolated spinal cord involvement was more frequent in non‐relapsed patients.

Markers reflecting BBB dysfunction showed pronounced group differences. Patients in the relapse group exhibited significantly higher QAlb values (median: 7.46 vs. 4.74, *p* < 0.001) and a higher proportion of elevated QAlb status (*p* = 0.001). Among CSF immune indices, QIgG and QIgA levels were higher in relapsed patients, whereas the IgG index and 24‐h intrathecal IgG synthesis did not differ significantly between groups.

Peripheral inflammatory activation was also more prominent in relapsed patients. Both NLR and MLR were significantly elevated (both *p* < 0.001), whereas PLR and CRP levels did not differ between groups. Relapsed patients further exhibited higher baseline EDSS scores (median: 4.0 vs. 3.0, *p* = 0.005), with a higher proportion of moderate‐to‐severe disability.

Maintenance treatment patterns differed significantly between groups (*p* = 0.041). Relapsed patients were more likely to receive no standardized maintenance therapy, while biologic immunotherapy was more frequently used in non‐relapsed patients. Acute attack treatment modalities did not differ significantly.

### 3.2 Visualization of Key Continuous Variables

**FIGURE 1:**
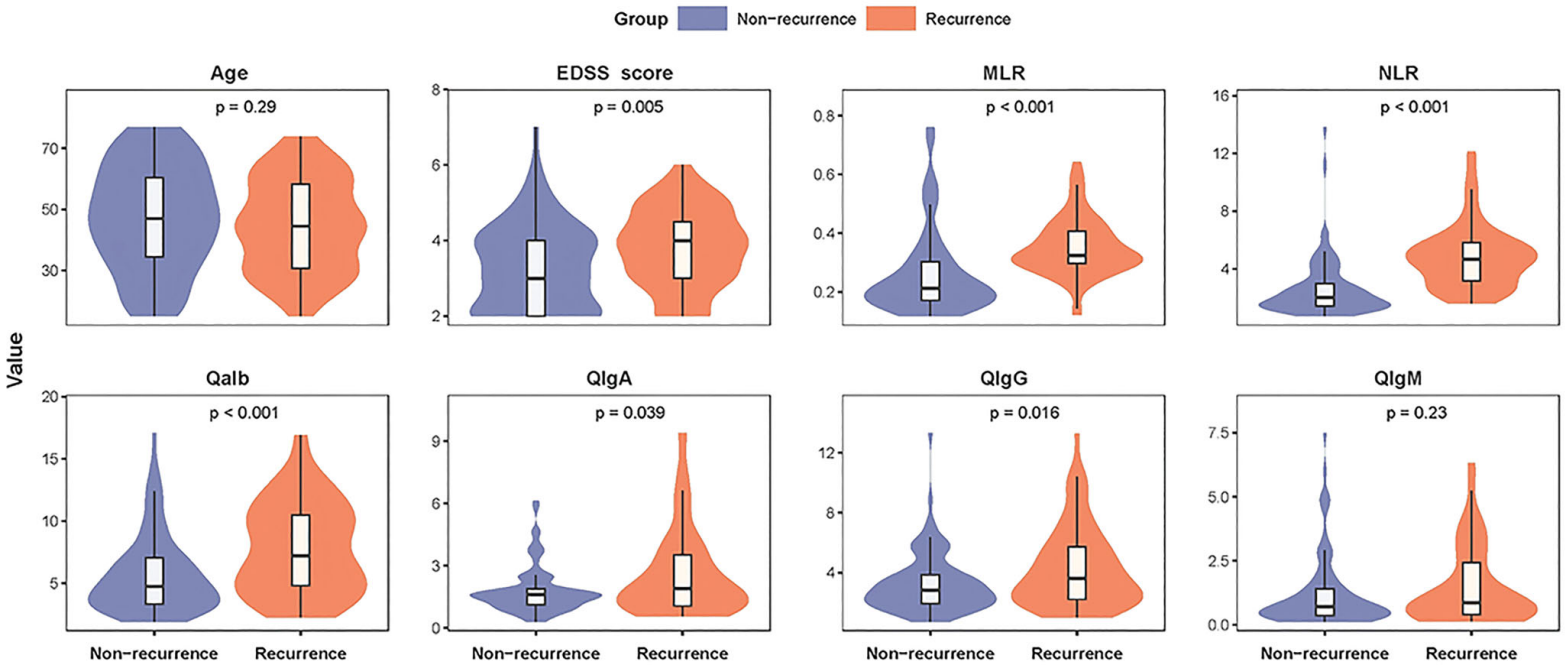
Distribution of baseline clinical, cerebrospinal fluid, and peripheral inflammatory variables in relapsed and non‐relapsed NMOSD patients. Violin plots illustrating the distribution of key baseline continuous variables in patients with neuromyelitis optica spectrum disorder (NMOSD) stratified by relapse status. Variables included age, Expanded Disability Status Scale (EDSS), cerebrospinal fluid/serum albumin quotient (QAlb), cerebrospinal fluid/serum immunoglobulin A quotient (QIgA), cerebrospinal fluid/serum immunoglobulin G quotient (QIgG), cerebrospinal fluid/serum immunoglobulin M quotient (QIgM), neutrophil‐to‐lymphocyte ratio (NLR), and monocyte‐to‐lymphocyte ratio (MLR). The width of each violin represents the kernel density of the data, with median and interquartile ranges indicated.

To illustrate group‐level differences in key continuous variables, violin plots were generated for age, EDSS score, QAlb, QIgA, QIgG, QIgM, NLR and MLR (Figure 1). Relapsed patients exhibited a significant rightward shift and broader distribution of QAlb (*p* < 0.001), NLR (*p* < 0.001), and MLR (*p* < 0.001), indicating more severe BBB dysfunction and systemic inflammatory activation.

The baseline EDSS score was significantly higher in the relapse group (*p* = 0.005). In contrast, age (*p* = 0.29) and QIgM (*p* = 0.23) showed no significant group differences. QIgA (*p* = 0.039) and QIgG (*p* = 0.016) exhibited modest distributional differences with substantial overlap, suggesting limited discriminative capacity.

### 3.3 Visualization of Clinical and Laboratory Characteristics

**FIGURE 2:**
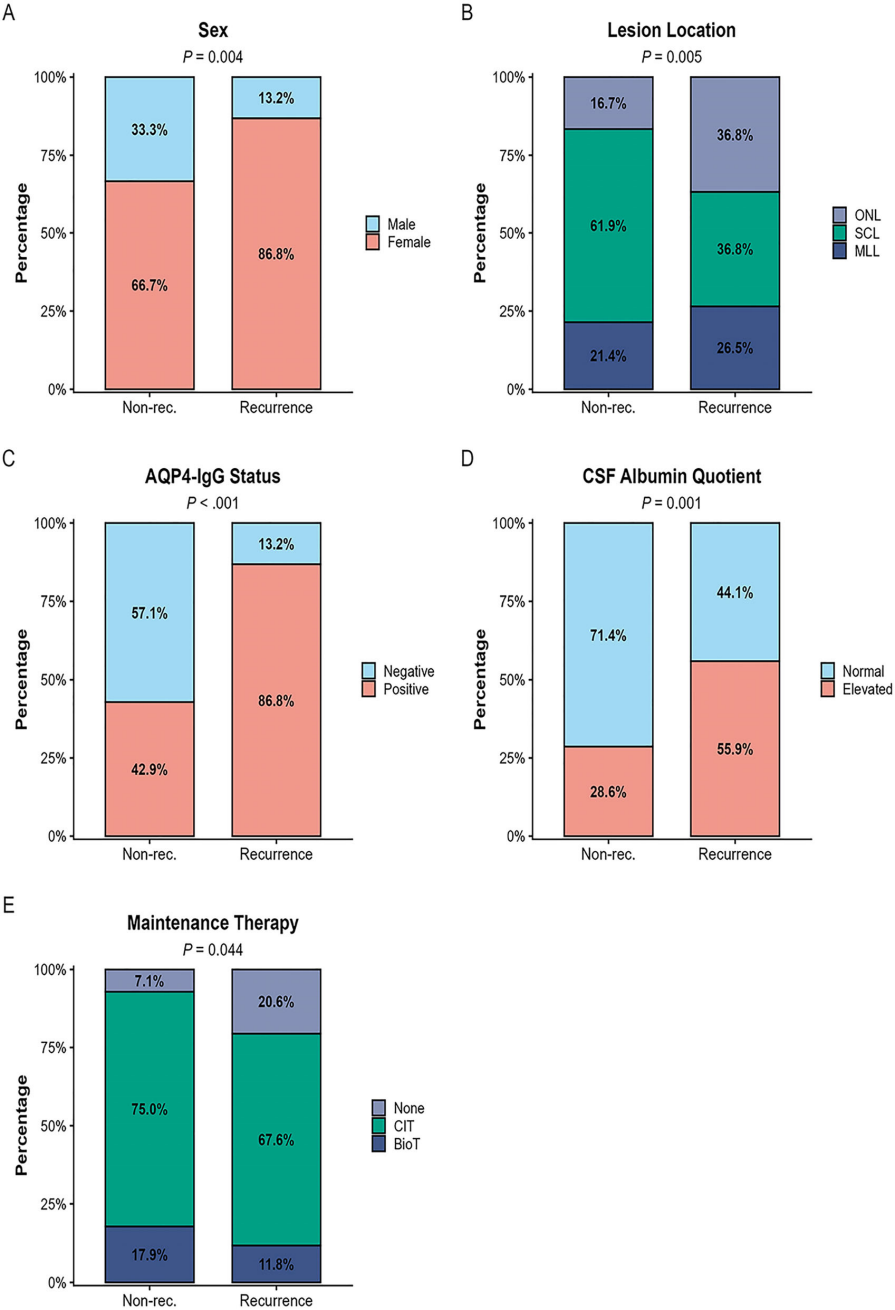
Baseline clinical characteristics of relapsed and non‐relapsed NMOSD patients. Comparison of baseline clinical characteristics between patients with neuromyelitis optica spectrum disorder (NMOSD) who experienced relapse and those who remained relapse‐free during follow‐up. Variables include sex, lesion distribution at onset (optic neuritis–predominant lesions, spinal cord lesions, or multifocal lesions), aquaporin‐4 immunoglobulin G (AQP4‐IgG) serostatus, baseline Expanded Disability Status Scale (EDSS) category, and maintenance therapy. Data are presented as proportions, and group differences were assessed using the *χ* ^2^ test or Fisher's exact test, as appropriate. Sex is categorized as male or female. Maintenance therapy is classified as no maintenance therapy, conventional immunosuppressive therapy, or biologic immunotherapy. Statistically significant differences between groups are indicated.

Categorical and selected clinical characteristics were visualized to further compare relapsed and non‐relapsed patients (Figure 2). The proportion of female patients was significantly higher in the relapse group (*p* = 0.004), and lesion location distribution differed significantly between groups (*p* = 0.005). Serum AQP4‐IgG positivity was strongly associated with relapse risk (*p* < 0.001). An elevated CSF albumin quotient was more prevalent in the relapse group (*p* = 0.001), supporting the presence of impaired BBB integrity. In addition, the maintenance therapy distribution differed significantly (*p* = 0.044), with a lower proportion of relapsed patients receiving biologic therapy (BioT) and a higher proportion receiving no maintenance therapy (NoMT).

### 3.4 Univariable Cox Regression Analysis of Relapse Risk

Univariable Cox proportional hazards regression was performed to identify factors associated with time to first relapse (Table 2). Female sex (HR: 2.56, 95% CI: 1.27–5.17, *p* = 0.009), serum AQP4‐IgG positivity (HR: 5.65, 95% CI: 2.80–11.43, *p* < 0.001), and indicators of BBB dysfunction, including elevated QAlb, were all significantly associated with an increased risk of relapse.

**TABLE 2:** Univariable Cox proportional hazards regression analysis for predictors of relapse in NMOSD.

Variable	HR	95% CI	*p*
Age	0.99	**0.98–1.01**	0.401
Location			
ONL	Reference	**—**	**—**
SCL	0.46	**0.26–0.80**	0.006
MLL	0.72	**0.39–1.32**	0.284
Sex	2.56	**1.27–5.17**	0.009
Serum AQP4 IgG	5.65	**2.80–11.43**	< 0.001
Qalb group	2.17	**1.34–3.51**	0.002
Qalb	1.13	**1.06–1.19**	< 0.001
QIgG	1.08	**1.01–1.15**	0.017
QIgA	1.1	**1.03–1.17**	0.003
QIgM	1.07	**1.01–1.14**	0.017
CSF IgG index	0.65	**0.28–1.47**	0.296
CSF IgG 24h	1.01	**0.99–1.02**	0.535
NLR	1.17	**1.10–1.23**	< 0.001
MLR	16.5	**4.70–57.97**	< 0.001
CRP	1	**0.97–1.02**	0.166
EDSS group	1.6	**1.00–2.58**	0.052
EDSS score	1.19	**1.00–1.41**	0.045
Acute attack therapy			
IVMP	Reference	**—**	**—**
IVIG	0.83	**0.46–1.50**	0.537
PE	0.77	**0.19–3.18**	0.723
Maintenance therapy			
NoMT	Reference	**—**	**—**
CIT	0.36	**0.18–0.71**	0.003
BioT	0.07	**0.02–0.23**	< 0.001

*Note*: Univariable Cox proportional hazards regression analysis evaluating associations between baseline demographic, clinical, cerebrospinal fluid, blood inflammatory, and treatment‐related variables and time to first relapse in patients with neuromyelitis optica spectrum disorder. The results are presented as hazard ratios (HRs) with 95% confidence intervals (CIs). A two‐sided *p* < 0.05 was considered statistically significant.

Abbreviations: AQP4‐IgG, aquaporin‐4 immunoglobulin G; BioT, biologic immunotherapy; CI, confidence interval; CIT, conventional immunosuppressive therapy; CRP, C‐reactive protein; CSF, cerebrospinal fluid; EDSS, Expanded Disability Status Scale; HR, hazard ratio; MLR, monocyte‐to‐lymphocyte ratio; NLR, neutrophil‐to‐lymphocyte ratio; NMOSD, neuromyelitis optica spectrum disorder; NoMT, no maintenance therapy; PLR, platelet‐to‐lymphocyte ratio; QAlb, cerebrospinal fluid/serum albumin quotient; QIgA, cerebrospinal fluid/serum immunoglobulin A quotient; QIgG, cerebrospinal fluid/serum immunoglobulin G quotient; QIgM, cerebrospinal fluid/serum immunoglobulin M quotient.

QAlb was strongly associated with relapse both as a categorical variable (HR: 2.17, 95% CI: 1.34–3.51; *p* = 0.002) and as a continuous variable (HR per unit increase 1.13; *p* < 0.001). To investigate whether BBB permeability was associated with baseline disease severity, we performed a correlation analysis between the QAlb and EDSS scores. A positive correlation was observed (Figure S2). Among peripheral inflammatory markers, both NLR (HR: 1.17 per unit increase; *p* < 0.001) and MLR (HR: 16.50, 95% CI: 4.70–57.97; *p* < 0.001) were significantly associated with relapse.

A higher baseline EDSS score was also associated with increased relapse risk (HR: 1.19; *p* = 0.045), whereas maintenance therapy, particularly biologic immunotherapy, was strongly protective (HR: 0.07; *p* < 0.001). Acute attack treatment modalities were not associated with relapse risk. The overall pattern of associations is summarized in Table 2 and visually presented in Figure S3.

### 3.5 Multivariable Cox Regression Analysis

**FIGURE 3:**
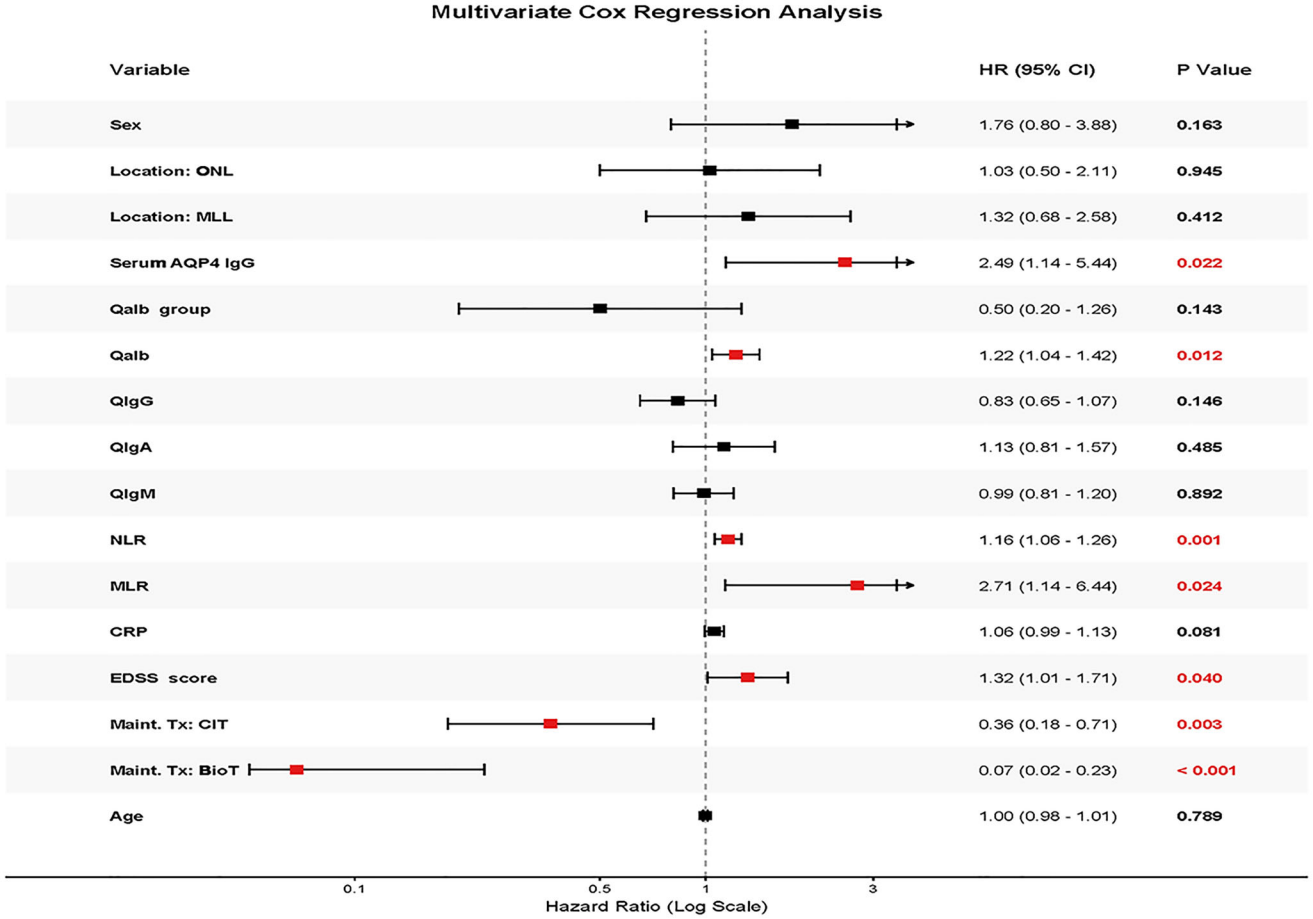
Multivariate Cox regression analysis of predictors for relapse in NMOSD patients. Forest plot illustrating the results of multivariate Cox proportional hazards regression analysis identifying independent predictors of relapse in patients with neuromyelitis optica spectrum disorder (NMOSD). The analysis included demographic variables (age, sex), clinical characteristics (disease location: optic neuritis [ON], myelitis [ML]; baseline Expanded Disability Status Scale [EDSS] score), laboratory parameters (serum AQP4‐IgG status, cerebrospinal fluid [CSF]/serum albumin quotient [QAlb], CSF/serum immunoglobulin quotients [QIgG, QIgA, QIgM], neutrophil‐to‐lymphocyte ratio [NLR], monocyte‐to‐lymphocyte ratio [MLR], C‐reactive protein [CRP]), and maintenance therapy types (conventional immunosuppressive therapy [CIT], biologic therapy [BioT]). The horizontal axis represents the hazard ratio (HR) on a logarithmic scale, with each horizontal line indicating the 95% confidence interval (95% CI) of the corresponding HR. A vertical dashed line at HR = 1 denotes no predictive effect; values to the right of the line indicate increased relapse risk, while values to the left indicate decreased risk. Statistical significance (*p* < 0.05) is denoted by HRs whose 95% CIs do not cross the dashed line. Independent predictors of increased NMOSD relapse risk included serum AQP4‐IgG positivity (HR = 2.49, 95% CI: 1.14–5.44, *p* = 0.022), elevated QAlb (HR = 1.22, 95% CI: 1.04–1.42, *p* = 0.012), higher NLR (HR = 1.16, 95% CI: 1.06–1.26, *p* = 0.001), higher MLR (HR = 2.71, 95% CI: 1.14–6.44, *p* = 0.024), and higher baseline EDSS score (HR = 1.32, 95% CI: 1.01–1.71, *p* = 0.040). In contrast, maintenance therapy (both CIT: HR = 0.36, 95% CI: 0.18–0.71, *p* = 0.003; BioT: HR = 0.07, 95% CI: 0.02–0.23, *p* < 0.001) was identified as a protective factor against relapse. These findings provide the statistical basis for constructing the relapse prediction nomogram (Figure 6) and support the clinical significance of targeting systemic inflammation and blood–brain barrier function in NMOSD management.

Variables with *p* < 0.10 in univariable analyses were entered into multivariable Cox proportional hazards regression models to identify independent predictors of relapse (Table 3). The adjusted HRs and 95% CIs of the final model are visually summarized in a forest plot (Figure 3).

**TABLE 3:** Multivariable Cox proportional hazards regression analysis for independent predictors of relapse in NMOSD.

Variable	HR	95% CI	*p*
Age	1.0	0.98–1.01	0.789
Maintenance therapy			
NoMT	Reference	—	—
CIT	0.36	0.18–0.71	0.003
BioT	0.07	0.02–0.23	< 0.001
Sex	1.76	0.80–3.88	0.163
Location			
ONL	Reference	—	—
SCL	0.97	0.47–2.01	0.945
MLL	1.29	0.64–2.62	0.481
Serum AQP4 IgG	2.49	1.14–5.44	0.022
Qalb group	0.5	0.20–1.26	0.143
Qalb	1.22	1.04–1.42	0.012
QIgG	0.83	0.65–1.07	0.146
QIgA	1.13	0.81–1.57	0.485
QIgM	0.99	0.81–1.20	0.892
NLR	1.16	1.06–1.26	0.001
MLR	2.71	1.14–6.44	0.024
CRP	1.06	0.99–1.13	0.081
EDSS score	1.32	1.01–1.71	0.04

*Note*: Multivariable Cox proportional hazards regression model including variables with *p* < 0.10 in univariable analyses to identify independent predictors of relapse in neuromyelitis optica spectrum disorder. The results are expressed as hazard ratios (HRs) with 95% confidence intervals (CIs). Proportional hazards assumptions were tested and met for all included variables.

Abbreviations: AQP4‐IgG, aquaporin‐4 immunoglobulin G; BioT, biologic immunotherapy; CI, confidence interval; CIT, conventional immunosuppressive therapy; CSF, cerebrospinal fluid; EDSS, Expanded Disability Status Scale; HR, hazard ratio; MLR, monocyte‐to‐lymphocyte ratio; NLR, neutrophil‐to‐lymphocyte ratio; NMOSD, neuromyelitis optica spectrum disorder; NoMT, no maintenance therapy; QAlb, cerebrospinal fluid/serum albumin quotient.

After adjustment for potential confounders, AQP4‐IgG positivity remained independently associated with relapse (HR: 2.49, 95% CI: 1.14–5.44; *p* = 0.022). QAlb analyzed as a continuous variable retained independent prognostic significance (HR per unit increase 1.22; *p* = 0.012), whereas categorical QAlb status did not remain significant, indicating a dose‐dependent relationship between BBB dysfunction and relapse risk.

Among peripheral inflammatory markers, both NLR (HR: 1.16 per unit increase; *p* = 0.001) and MLR (HR: 2.71, 95% CI: 1.14–6.44; *p* = 0.024) independently predicted relapse. As illustrated in Figure 3, MLR exhibited one of the largest effect sizes among continuous inflammatory variables.

Baseline EDSS score and maintenance therapy remained independently associated with relapse risk, with biologic immunotherapy showing a strong protective effect. In contrast, sex, lesion location, CSF immunoglobulin indices, and CRP were not independently associated with relapse after multivariable adjustment.

### 3.6 Subgroup Analysis According to AQP4‐IgG Serostatus

Given that AQP4‐IgG seropositivity is a well‐established determinant of relapse risk in NMOSD, we further performed subgroup analyses to evaluate whether the proposed model retained its prognostic value beyond serological status.

Kaplan‒Meier analysis demonstrated that AQP4‐IgG seropositive patients had a significantly higher risk of relapse than seronegative patients (log rank *p* < 0.01; Figure S4), confirming the clinical relevance of AQP4 serostatus in our cohort.

To further assess the incremental prognostic value of the model, we applied the same modeling framework in the AQP4‐IgG seropositive subgroup. Multivariable Cox proportional hazards regression was reperformed in this subgroup, and the results were visualized using a forest plot (Figure S5). In this subgroup, key predictors identified in the overall cohort, including QAlb and peripheral inflammatory markers (NLR and MLR), remained significantly associated with relapse risk. The integrated model demonstrated acceptable discriminative performance, with a 12‐month time‐dependent AUC of 0.804 and a corrected *C*‐index of 0.711, along with good calibration and favorable clinical utility, as shown in Figure S6.

In addition, exploratory analyses were conducted in the subset of patients with available quantitative AQP4‐IgG titers (*n* = 62). Patients with higher antibody titers exhibited an increased risk of relapse in univariable Cox regression analysis (HR: 2.66, 95% CI: 1.34–5.28; *p* = 0.005), suggesting a dose–response relationship between antibody burden and relapse susceptibility (Table S1 and Figure S7).

Furthermore, interaction analyses between AQP4 serostatus and key predictors (including QAlb, NLR, and MLR) were performed. No statistically significant interactions were observed (all *p* for interaction > 0.05), indicating that AQP4 serostatus did not significantly modify the effects of these variables on relapse risk (Figure S8).

Collectively, these findings indicate that the integrated model provides additional prognostic value beyond AQP4 serostatus and remains applicable in clinically relevant AQP4‐IgG seropositive patients.

Due to the relatively limited sample size of seronegative patients, detailed subgroup modeling in this population was not performed.

### 3.7 Development and Comparison of Predictive Models

Based on independent predictors identified in the multivariable analysis, three hierarchical prognostic models were constructed: a clinical model, a clinical plus CSF model, and an integrated clinical plus CSF plus blood inflammatory marker model.

**FIGURE 4:**
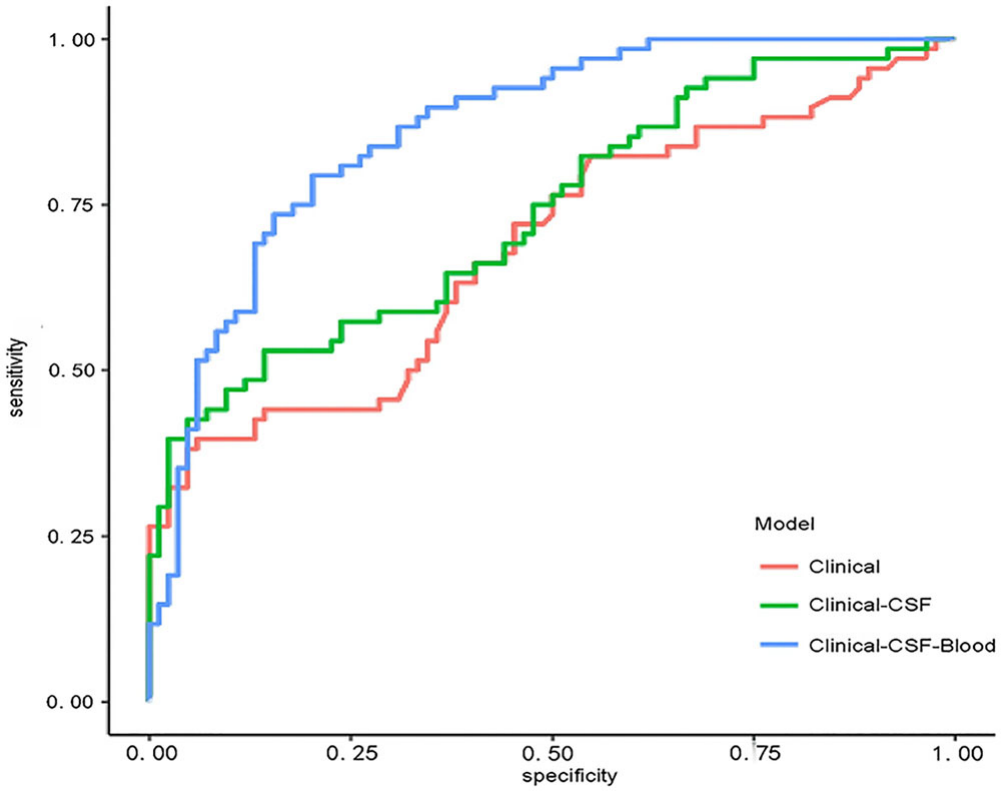
Receiver operating characteristic (ROC) curves for hierarchical prognostic models. ROC curves comparing the discriminative performance of three Cox regression–based models for relapse prediction: a clinical model, a clinical plus cerebrospinal fluid (CSF) model, and an integrated clinical–CSF–blood inflammatory model. Model performance was quantified using the area under the ROC curve (AUC).

Model discrimination improved stepwise with the sequential inclusion of CSF indices and peripheral inflammatory markers. The clinical model yielded an AUC of 0.693, indicating modest discrimination. Incorporation of CSF parameters improved the AUC to 0.744, while the fully integrated model achieved the highest discrimination, with an AUC of 0.850 (Figure 4).

### 3.8 Time‐Dependent ROC Analysis

**FIGURE 5:**
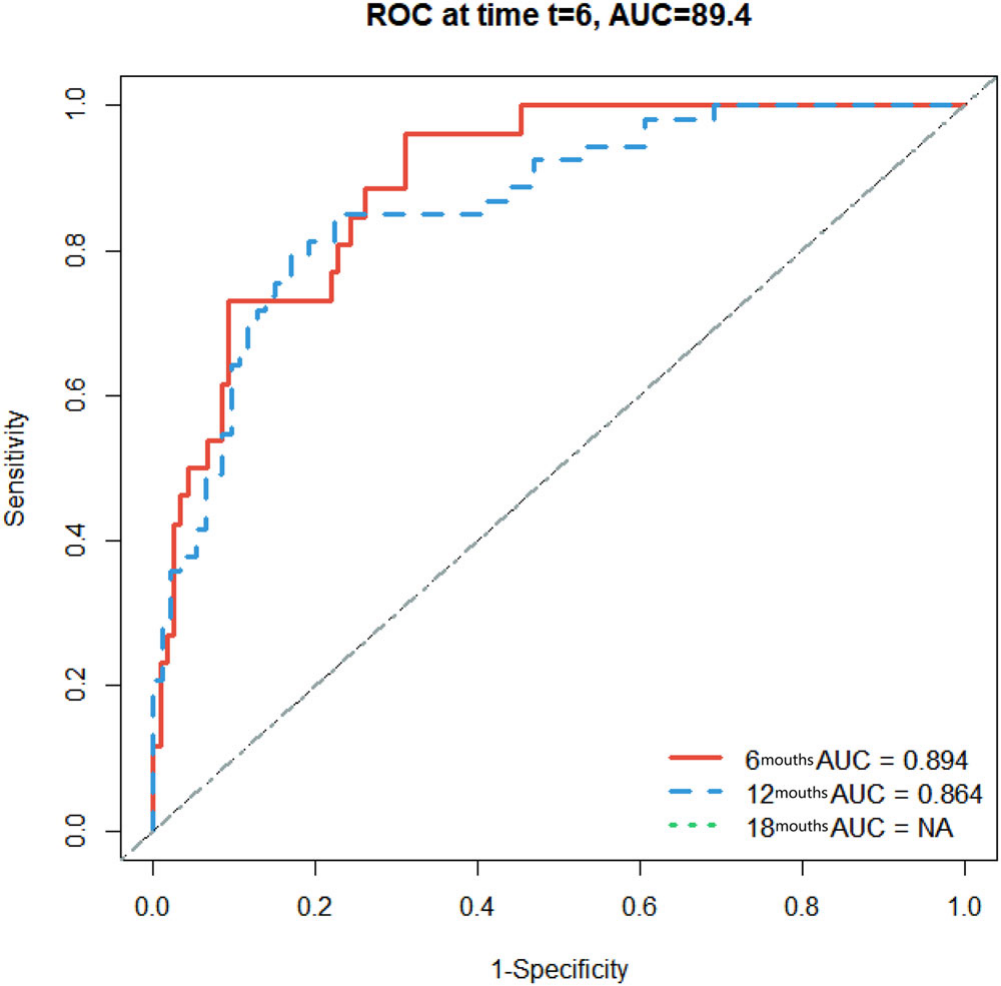
Time‐dependent receiver operating characteristic (ROC) analysis of the integrated prognostic model. Time‐dependent ROC curves evaluating the predictive accuracy of the integrated clinical–cerebrospinal fluid–blood inflammatory model at 6 and 12 months. The area under the curve (AUC) at each time point reflects model discrimination for relapse risk over time.

Time‐dependent ROC analysis demonstrated excellent early predictive performance of the integrated model. The time‐dependent AUC was 0.894 at 6 months and sustained discrimination at 12 months (AUC: 0.864) (Figure 5). Reliable estimation beyond 12 months was not feasible due to the limited number of patients remaining at risk.

### 3.9 Nomogram Construction and Internal Validation

**FIGURE 6:**
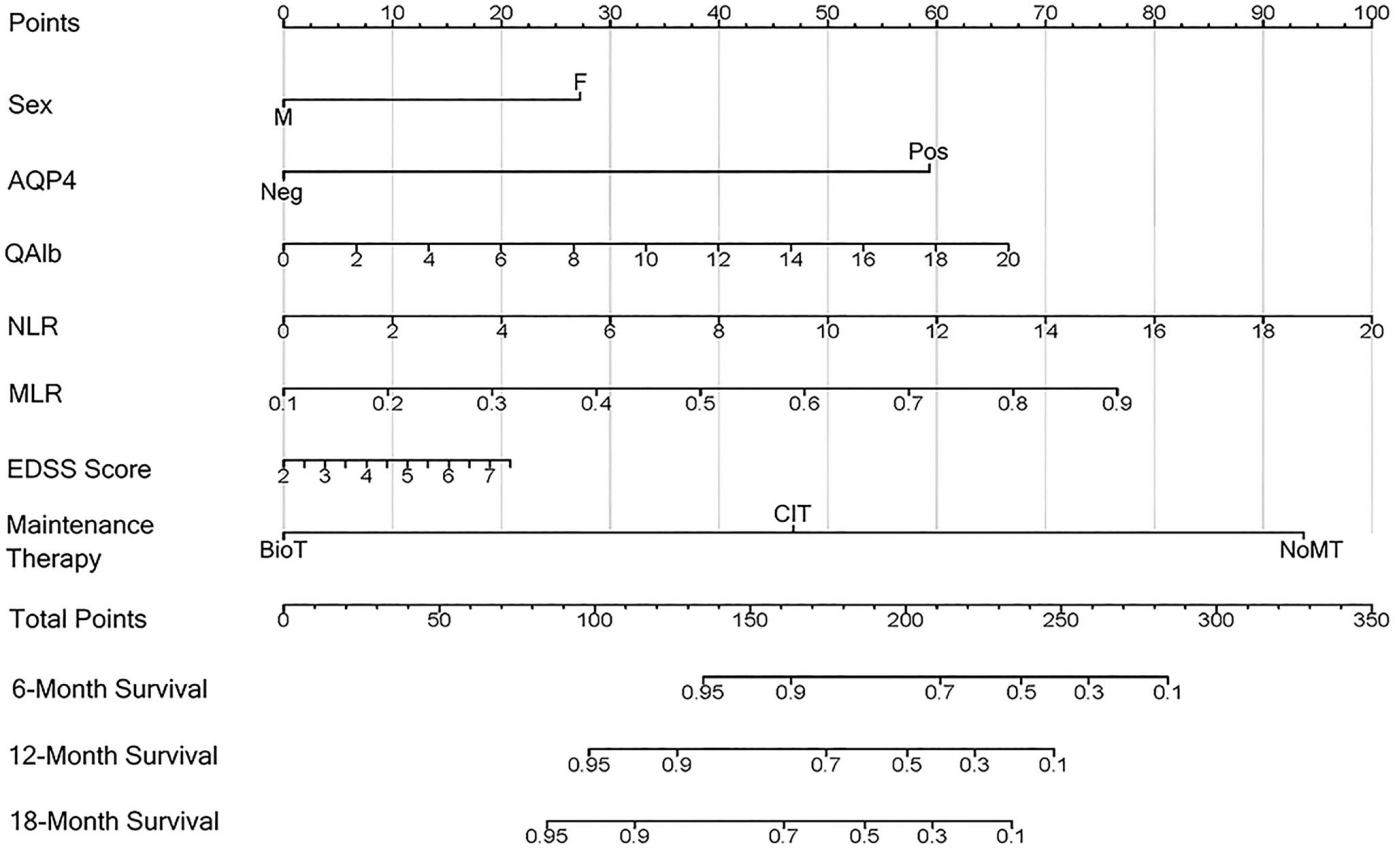
Nomogram for individualized relapse risk prediction in NMOSD. A Cox regression–based nomogram integrating sex, aquaporin‐4 immunoglobulin G (AQP4‐IgG) serostatus, cerebrospinal fluid/serum albumin quotient (QAlb), neutrophil‐to‐lymphocyte ratio (NLR), monocyte‐to‐lymphocyte ratio (MLR), baseline Expanded Disability Status Scale (EDSS), and maintenance therapy to estimate individualized probabilities of relapse at 6, 12, and 18 months. For categorical variables, sex is coded as male (M) or female (F); AQP4 serostatus is coded as negative (Neg) or positive (Pos); and maintenance therapy is categorized as no maintenance therapy (NoMT), conventional immunosuppressive therapy (CIT), or biologic immunotherapy (BioT).

A nomogram was constructed based on the final multivariable Cox model to estimate individualized relapse probabilities at 6, 12, and 18 months (Figure 6). Internal validation using 1000 bootstrap resamples yielded an optimism‐corrected *C*‐index of 0.811, indicating good discrimination and model stability.

**FIGURE 7:**
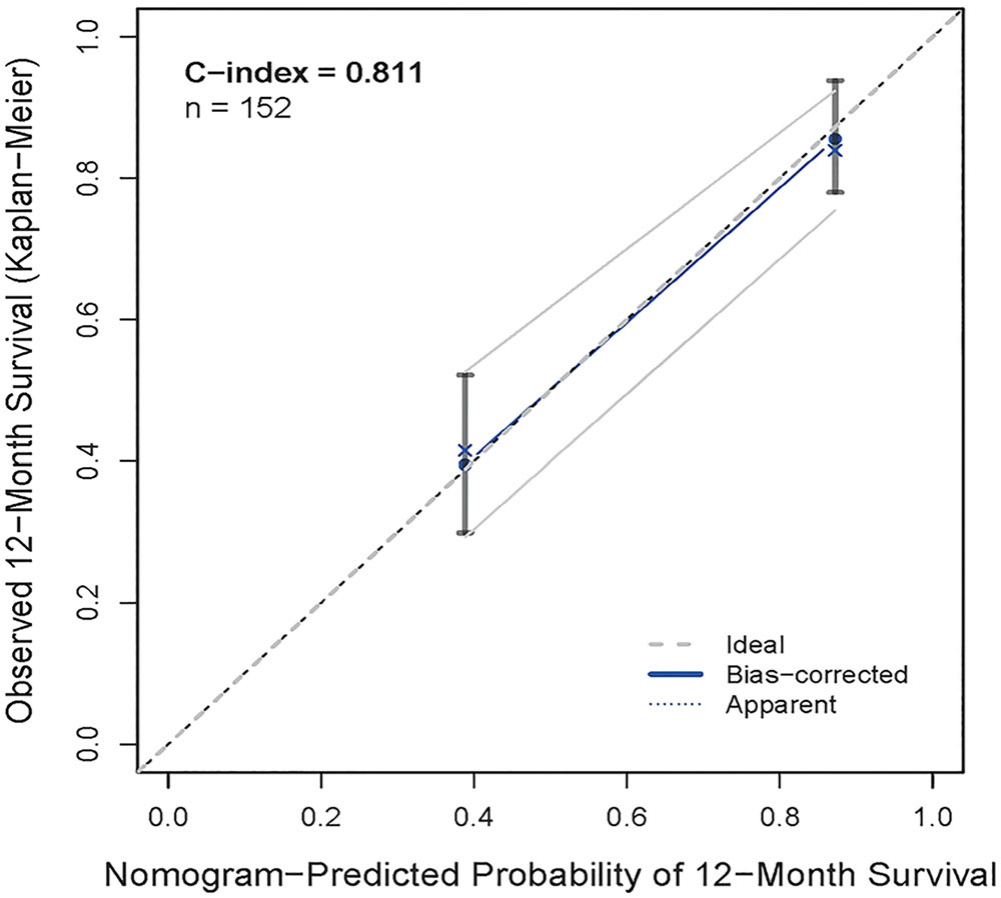
Calibration of the nomogram for 12‐month relapse prediction. Calibration plot comparing predicted versus observed 12‐month relapse probabilities derived from the nomogram. The dashed diagonal line represents perfect agreement between the prediction and observation. Calibration was assessed using bootstrap resampling (1,000 iterations).

Calibration analysis at 12 months showed good agreement between predicted and observed relapse probabilities, with calibration curves closely approximating the ideal 45‐degree line (Figure 7).

### 3.10 DCA

**FIGURE 8:**
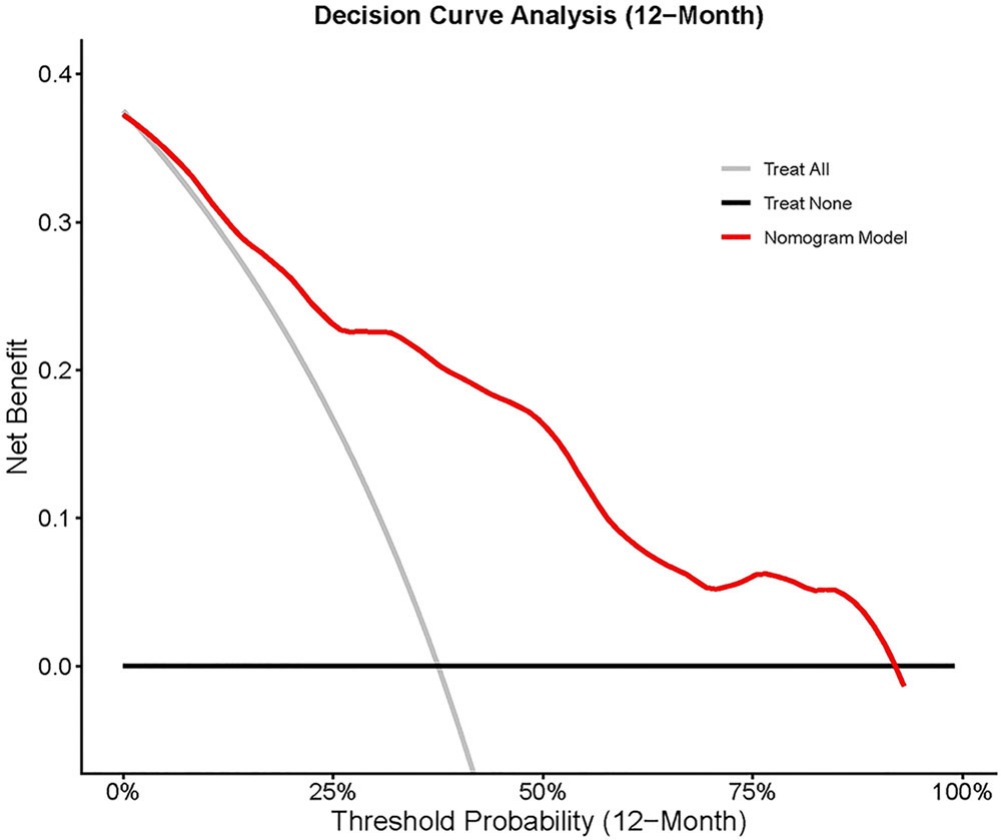
Decision curve analysis for the nomogram at 12 months. Decision curve analysis (DCA) evaluating the clinical utility of the nomogram for 12‐month relapse prediction. The y‐axis represents the net benefit, and the x‐axis represents the threshold probability. The nomogram is compared with “treat‐all” and “treat‐none” strategies across a range of clinically relevant threshold probabilities.

DCA at 12 months demonstrated that the nomogram consistently provided higher net clinical benefit than both treat‐all and treat‐none strategies across a wide range of threshold probabilities (0%–95%) (Figure 8). The net benefit advantage was most pronounced at lower threshold probabilities (0%–50%), corresponding to common clinical decision‐making scenarios. Only when the threshold probability approached 100% did the net benefit converge toward the treat‐none strategy.

## 4 Discussion

In this retrospective cohort study of 152 patients with NMOSD, we demonstrate that baseline BBB dysfunction, reflected by elevated QAlb, and peripheral systemic inflammatory activation, reflected by increased NLR and MLR, are independently and additively associated with relapse risk. By integrating these biologically complementary domains with key clinical variables, we developed and internally validated a Cox regression‐based nomogram with robust discrimination, good calibration, and meaningful clinical net benefit.

A central finding of this study is that BBB vulnerability is not merely a secondary epiphenomenon of tissue injury but a determinant of relapse susceptibility in NMOSD. AQP4‐IgG antibodies are pathogenic only after gaining access to astrocytic end‐feet within the CNS, a process that critically depends on BBB disruption (Lennon et al. 2005; Saadoun et al. 2010; Bradl et al. 2009; Lucchinetti et al. 2002). Elevated QAlb, a widely accepted surrogate of BBB permeability, therefore represents a permissive state that facilitates the entry of circulating antibodies, complement components, and immune cells. Previous studies have associated elevated QAlb with disease severity and CSF inflammatory abnormalities in NMOSD (Reiber and Peter 2001; Jarius et al. 2011; Jarius and Wildemann 2013); our findings extend this evidence by demonstrating its independent prognostic relevance for future relapse. Importantly, this finding suggests that BBB vulnerability in NMOSD should be viewed not only as a consequence of established neuroinflammation but also as a predisposing biological state that lowers the threshold for subsequent antibody‐mediated CNS injury. BBB permeability can be influenced by several clinical factors, including age, medication exposure, and systemic infections. To minimize potential confounding factors, patients with acute infections were excluded during cohort enrollment. In addition, age and treatment‐related variables were incorporated into the Cox regression models to further adjust for these influences. These methodological considerations strengthen the robustness of the observed association between QAlb and relapse risk.

In parallel, peripheral inflammatory ratios capture a systemic shift toward innate immune predominance. Elevated NLR and MLR have been linked to disease activity and relapse risk in NMOSD and other autoimmune disorders (Wu et al. 2025; Fang et al. 2023). Consistent with our findings, both NLR and MLR have been shown to predict subsequent disability accumulation, including EDSS scores at one‐year follow‐up, even in pediatric NMOSD populations, suggesting that these inflammatory signatures may reflect a common disease biology across age groups (Devlin and Gombolay 2023). Activated neutrophils and monocytes release pro‐inflammatory cytokines, proteases, and matrix metalloproteinases that can compromise endothelial tight junctions and further destabilize BBB integrity (Rempe et al. 2016; Galea 2021; Lee et al. 2025). Collectively, these observations support a model in which peripheral innate immune activation acts upstream of central immune injury by modulating endothelial integrity and amplifying BBB vulnerability before overt CNS immune infiltration.

**FIGURE 9:**
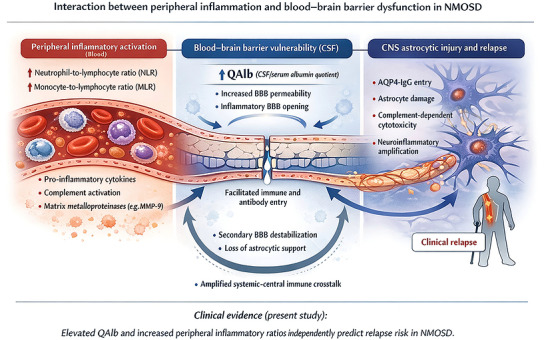
Proposed mechanistic framework linking blood–brain barrier dysfunction and systemic inflammation to relapse susceptibility in NMOSD. Schematic illustration of the proposed pathophysiological interplay between blood–brain barrier (BBB) dysfunction and peripheral systemic inflammation in neuromyelitis optica spectrum disorder (NMOSD). An elevated cerebrospinal fluid/serum albumin quotient (QAlb) reflects increased BBB permeability, facilitating central nervous system entry of circulating aquaporin‐4 immunoglobulin G (AQP4‐IgG), complement components, and activated immune cells. Concurrently, systemic innate immune activation, reflected by an increased neutrophil‐to‐lymphocyte ratio (NLR) and monocyte‐to‐lymphocyte ratio (MLR), promotes endothelial dysfunction and further destabilizes BBB integrity. Astrocytic injury and complement activation amplify local neuroinflammation, creating a self‐reinforcing loop that increases relapse susceptibility.

Importantly, our statistical findings align with a coherent mechanistic framework in which BBB dysfunction and systemic inflammation form a self‐reinforcing pathological loop (Figure 9). Although the present study does not directly interrogate molecular signaling pathways at the experimental level, the proposed framework is grounded in well‐established neuroimmunological mechanisms linking endothelial dysfunction, astrocytopathy, and complement‐mediated injury in NMOSD. In this context, our model should be interpreted as a clinically anchored, mechanism‐informed synthesis that integrates central barrier vulnerability and systemic immune activation rather than as a claim of novel molecular pathway discovery. As illustrated, baseline BBB vulnerability permits CNS entry of pathogenic AQP4‐IgG and complement, triggering astrocytic injury and local immune amplification. This central injury further destabilizes BBB integrity, while heightened peripheral inflammation amplifies endothelial damage and immune cell recruitment. This dynamic interplay provides a biologically plausible explanation for the independent and additive prognostic effects observed in our multivariable model.

In addition to the primary analyses, we performed several supplementary analyses to further clarify the role of AQP4‐IgG serostatus. Consistent with the current understanding, AQP4‐IgG seropositive patients exhibited a higher risk of relapse, confirming its role as a major disease‐defining and prognostic factor in NMOSD.

Importantly, subgroup analysis demonstrated that the proposed model retained its predictive performance in AQP4‐IgG seropositive patients. This finding is clinically meaningful, as seropositive patients are generally considered at high risk and are often treated with relapse‐prevention therapies regardless of baseline stratification. The ability of the model to further discriminate relapse risk within this high‐risk population suggests that it provides incremental prognostic value beyond serological status alone.

Furthermore, exploratory analyses indicated that higher AQP4‐IgG antibody titers were associated with an increased risk of relapse, supporting a potential dose–response relationship between antibody burden and disease activity. This observation is biologically plausible given the pathogenic role of AQP4‐IgG in mediating complement‐dependent astrocyte injury.

Notably, interaction analyses did not demonstrate significant effect modification by AQP4 serostatus, indicating that the associations of BBB dysfunction and systemic inflammatory activation with relapse risk are broadly consistent across serological subtypes. Taken together, these findings support the robustness and generalizability of the proposed model.

The mechanistic framework depicted in Figure 9 also explains the superior performance of the integrated clinical‐CSF‐blood inflammatory model. Incorporation of BBB integrity and systemic inflammatory markers yielded substantial gains in discrimination (AUC: 0.850 vs. 0.693 for the clinical‐only model), with stable internal validation and favorable DCA across clinically relevant thresholds. These findings highlight the value of integrating central and peripheral immune dimensions into relapse risk prediction. The stepwise improvement in model performance observed in our analyses mirrors this biological hierarchy, whereby clinical features capture disease phenotype, CSF indices reflect central barrier permissiveness, and blood inflammatory ratios represent upstream systemic immune pressure.

These results are consistent with and extend previous studies emphasizing the role of BBB disruption in NMOSD pathogenesis (Wingerchuk et al. 2015; Jarius and Wildemann 2013; Popescu et al. 2011; Hillmer et al. 2023) and the prognostic relevance of peripheral inflammatory markers such as NLR and MLR (Fang et al. 2023; Wang et al. 2023). Notably, few prior investigations have integrated these domains within a time‐to‐event framework. By combining CSF and peripheral biomarkers into a validated prognostic model, our study advances beyond descriptive associations toward individualized risk stratification, in line with contemporary recommendations for prognostic research in neuroinflammation (Riley et al. 2020).

Notably, the prognostic role of peripheral inflammatory ratios in NMOSD has not been entirely consistent across studies. For example, Carnero Contentti et al. reported that although the NLR was elevated in AQP4‐IgG‐positive NMOSD patients, it was not independently associated with clinical or imaging outcomes (Carnero Contentti et al. 2021). Several factors may contribute to this discrepancy. First, differences in study design may influence the results, as their study focused on NLR measured at disease onset in treatment‐naïve patients, whereas our analysis incorporated multiple inflammatory markers together with CSF indicators and clinical variables within a longitudinal relapse‐prediction framework. Second, differences in patient populations may also play a role; their cohort consisted exclusively of Latin American AQP4‐IgG‐positive NMOSD patients, whereas our cohort included an Asian population with both seropositive and seronegative cases. Third, variation in disease phase at sample collection may affect inflammatory biomarker performance, since laboratory parameters in our study were obtained during routine clinical evaluation rather than exclusively at the first untreated attack. Finally, the integrated multivariable modeling strategy used in our study may better capture the complex interaction between systemic inflammation and CNS vulnerability than single‐biomarker analyses. Taken together, these methodological and population differences may partly explain the apparent discrepancies and highlight the importance of context when interpreting inflammatory biomarkers in NMOSD.

From a clinical perspective, these findings have direct therapeutic implications. Patients exhibiting both BBB dysfunction and heightened systemic inflammation may represent a subgroup with increased vulnerability to antibody penetration and immune amplification. Such patients may benefit from closer monitoring and earlier escalation of high‐efficacy maintenance therapy. Several approved therapies for NMOSD target pathways directly relevant to this framework, including B‐cell depletion, complement inhibition, and interleukin‐6 (IL‐6) receptor blockade, which may modulate both systemic inflammation and barrier integrity (Yamamura et al. 2019; Traboulsee et al. 2020; Fung and Shirley 2023; Pittock et al. 2023; Pittock et al. 2019). Recent immunophenotyping studies have demonstrated that IL‐6 receptor blockade induces long‐term modulation of neutrophils and regulatory lymphocyte populations in NMOSD, providing mechanistic support for the observed interaction between systemic inflammation and CNS vulnerability (Matsuoka et al. 2024). In addition, circulating IL‐6 levels have been shown to correlate with clinical disease activity in NMOSD, further supporting the central role of inflammatory amplification loops in relapse susceptibility (Haramati et al. 2022). In this context, the proposed nomogram may serve not only as a prognostic tool but also as a framework to support mechanism‐informed treatment decisions.

This study has limitations, including its retrospective single‐center design, moderate sample size, baseline‐only biomarker assessment, and heterogeneous treatment exposure. In addition, quantitative anti‐AQP4‐IgG titers were available only for a subset of patients because earlier laboratory records reported antibody results qualitatively rather than quantitatively. Consequently, antibody titer analysis should be regarded as exploratory and may be subject to selection bias. External validation and prospective studies with longitudinal biomarker profiling are warranted. Nevertheless, all included variables are readily obtainable in routine clinical practice, supporting the translational potential of the model. Future studies incorporating longitudinal biomarker profiling, endothelial or astrocytic molecular signatures, and functional assessments of BBB permeability will be critical to directly validate the causal pathways proposed in this framework. Such approaches may further bridge clinical risk stratification with experimental neuroinflammation research, enabling refinement of mechanism‐based therapeutic targeting in NMOSD.

## 5 Conclusion

Our findings indicate that relapse susceptibility in NMOSD is driven by the convergence of BBB vulnerability and systemic inflammatory activation. Integration of these complementary dimensions into a prognostic nomogram enables individualized risk prediction and provides a biologically grounded framework for risk‐adapted management.

Importantly, the model demonstrated predictive value beyond AQP4‐IgG serostatus, supporting its applicability in clinically relevant patient populations. Exploratory analyses further suggested that higher anti‐AQP4‐IgG antibody titers may be associated with an increased risk of relapse; however, this observation requires validation in larger prospective cohorts with standardized quantitative measurements.

## Author Contributions

**Hao Yin**: data curation, investigation, validation, writing – review and editing. **Jing Shi**: conceptualization, writing – original draft, methodology, formal analysis, data curation, software, visualization. **Quanhua Zhai**: investigation, validation, data curation, writing – review and editing. **Cuiping Si**: investigation, validation, writing – review and editing, data curation. **Lei Zhang**: investigation, validation, writing – review and editing, data curation. **Huakun Liu**: conceptualization, funding acquisition, writing – review and editing, project administration, supervision, resources. **Huiqun Song**: investigation, validation, data curation, writing – review and editing. **Yuzhi Li**: investigation, validation, writing – review and editing, data curation. **Xingyue Zheng**: conceptualization, methodology, data curation, writing – original draft, formal analysis, software, visualization. **Jianwen Chen**: investigation, validation, writing – review and editing, data curation. **Chunbo Dong**: conceptualization, funding acquisition, supervision, project administration, writing – review and editing, resources. **Peixue Li**: investigation, validation, writing – review and editing, data curation. All the authors have read and approved the final manuscript.

## Funding

This work was supported by grants from the Key R&D Program of Jining (Grant number 2023YXNS105) and the Medical and Health Science and Technology Development Project of Shandong Province (Grant number 202303070266).

## Ethics Statement

This study was conducted in accordance with the Declaration of Helsinki and approved by the Ethics Committee of Jining No. 1 People's Hospital (KYLL‐202307‐102). For minors or incapacitated patients included in the study (if applicable), written informed consent was obtained from their legal guardians before data collection. All procedures involving human participants were strictly compliant with ethical and regulatory requirements.

## Consent

This manuscript is original, has not been published previously, and has not been submitted for publication elsewhere. All authors have read and approved the final version of the manuscript and consented to its submission. The authors agree to the publication of this work and confirm that all data and materials included in the manuscript are original or properly cited.

## Conflicts of Interest

The authors declare no conflicts of interest.

## Supporting information




**Supplementary Figure S1**. Flowchart of patient enrollment and follow‐up (detailed steps of screening, exclusion, and outcome assessment).

**Figure S2**. Correlation between cerebrospinal fluid/serum albumin quotient (QAlb) and Expanded Disability Status Scale (EDSS) score at baseline in NMOSD patients.

**Figure S3**. Forest plot of univariable Cox proportional hazards regression analysis for relapse risk in NMOSD.

**Figure S4**. Kaplan–Meier curves of relapse‐free survival in patients with AQP4‐IgG positive vs. negative status.

**Figure S5**. Subgroup multivariable Cox regression analysis in AQP4‐IgG seropositive patients.

**Figure S6**. Performance and clinical utility of the prognostic model for relapse risk in NMOSD.

**Figure S7**. Hazard ratio for disease relapse by AQP4‐IgG titer.

**Figure S8**. Subgroup hazard ratios for disease relapse: interaction analysis between AQP4 status and inflammatory biomarkers.

**Supplementary Table S1**. Univariate Cox regression analysis of the association between AQP4‐IgG titer and relapse risk.

## Data Availability

The datasets used and/or analyzed during the current study are available from the corresponding author upon reasonable request.
