# Supplementary material for: Integrated Blood Inflammatory Ratios and Cerebrospinal Fluid Blood‒Brain Barrier Dysfunction Predict Relapse Risk in Neuromyelitis Optica Spectrum Disorder
Source: Brain Behav. 2026 Jun 12;16(6):e71481. doi: 10.1002/brb3.71481 (PMC13263635; doi:10.1002/brb3.71481)

**Supplementary Figure S1: Flowchart of patient enrollment and follow-up (detailed steps of screening, exclusion, and outcome assessment).**


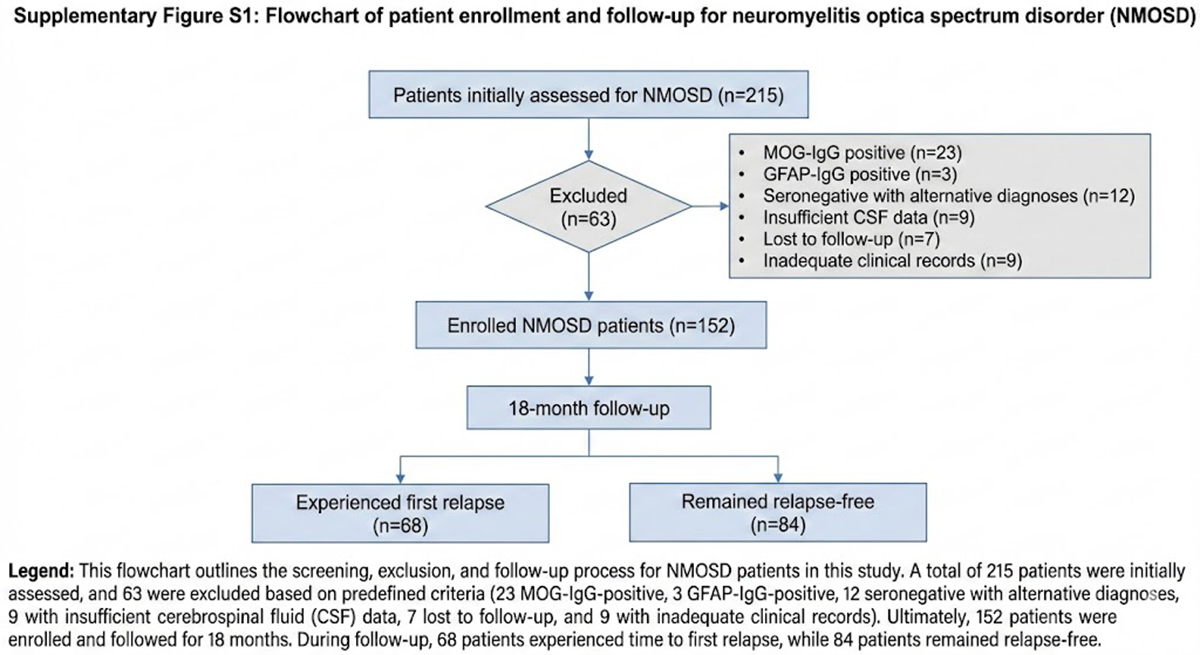

Supplement: Supplementary file 1 — Supplementary Figure S1. Flowchart of patient enrollment and follow‐up (detailed steps of screening, exclusion, and outcome assessment). [file BRB3-16-e71481-s009.docx]
