# Supplementary material for: Integrated Blood Inflammatory Ratios and Cerebrospinal Fluid Blood‒Brain Barrier Dysfunction Predict Relapse Risk in Neuromyelitis Optica Spectrum Disorder
Source: Brain Behav. 2026 Jun 12;16(6):e71481. doi: 10.1002/brb3.71481 (PMC13263635; doi:10.1002/brb3.71481)
Supplement: Supplementary file 2 — Figure S2. Correlation between cerebrospinal fluid/serum albumin quotient (QAlb) and Expanded Disability Status Scale (EDSS) score at baseline in NMOSD patients. [file BRB3-16-e71481-s004.docx]

**Figure S2：Correlation between cerebrospinal fluid/serum albumin quotient (QAlb) and Expanded Disability Status Scale (EDSS) score at baseline in NMOSD patients.**


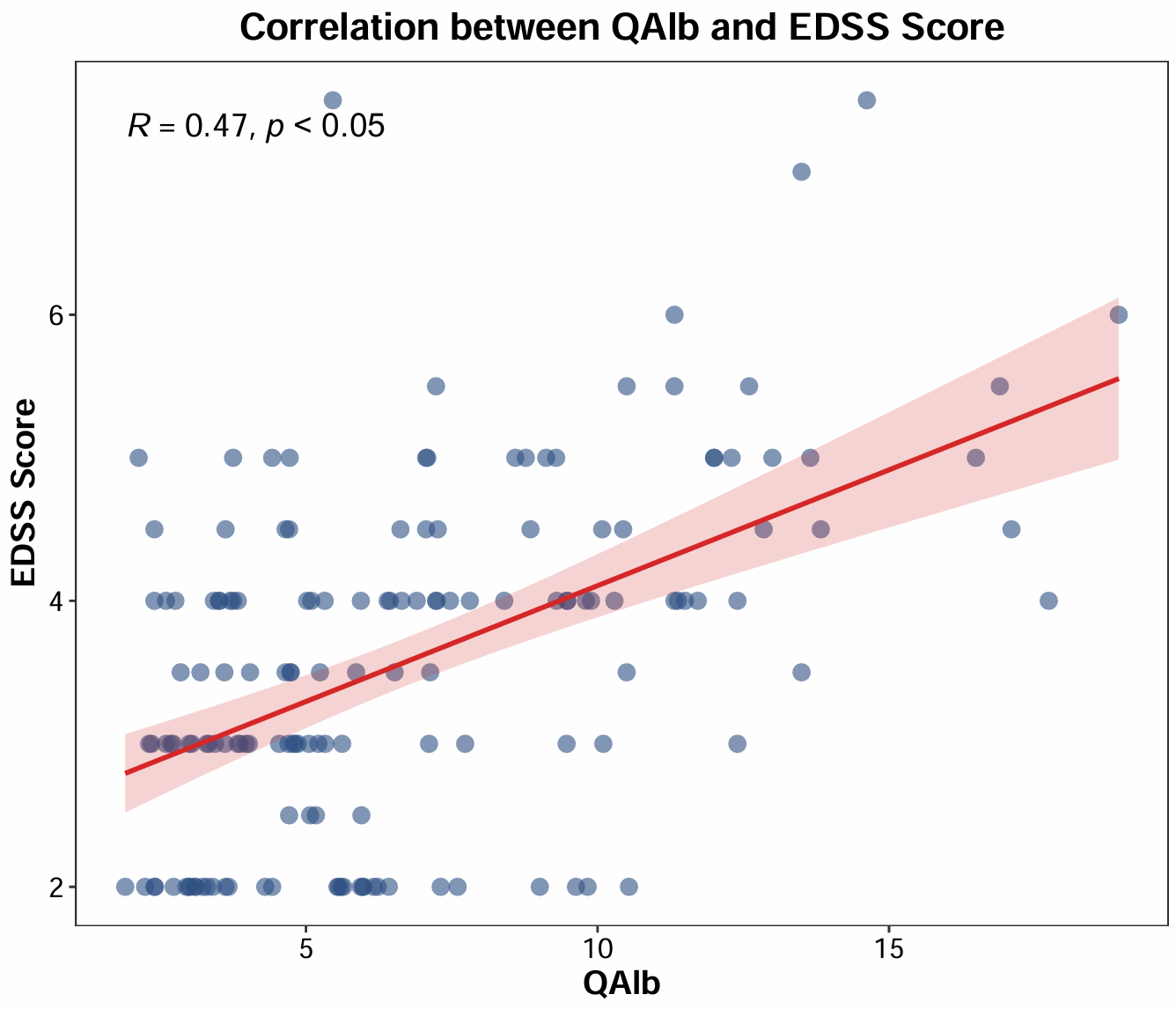


*Scatter plot showing the positive correlation between QAlb and EDSS score. The red line represents the linear regression fit, with the shaded area indicating the 95% confidence interval. Pearson correlation analysis revealed a significant positive association (R=0.47, p<0.05), suggesting that increased blood–brain barrier permeability, as reflected by higher QAlb, is associated with greater neurological disability during the acute phase of NMOSD.*
