# Supplementary material for: Integrated Blood Inflammatory Ratios and Cerebrospinal Fluid Blood‒Brain Barrier Dysfunction Predict Relapse Risk in Neuromyelitis Optica Spectrum Disorder
Source: Brain Behav. 2026 Jun 12;16(6):e71481. doi: 10.1002/brb3.71481 (PMC13263635; doi:10.1002/brb3.71481)
Supplement: Supplementary file 3 — Figure S3. Forest plot of univariable Cox proportional hazards regression analysis for relapse risk in NMOSD. [file BRB3-16-e71481-s008.docx]

**Figure S3: Forest plot of univariable Cox proportional hazards regression analysis for relapse risk in NMOSD.**


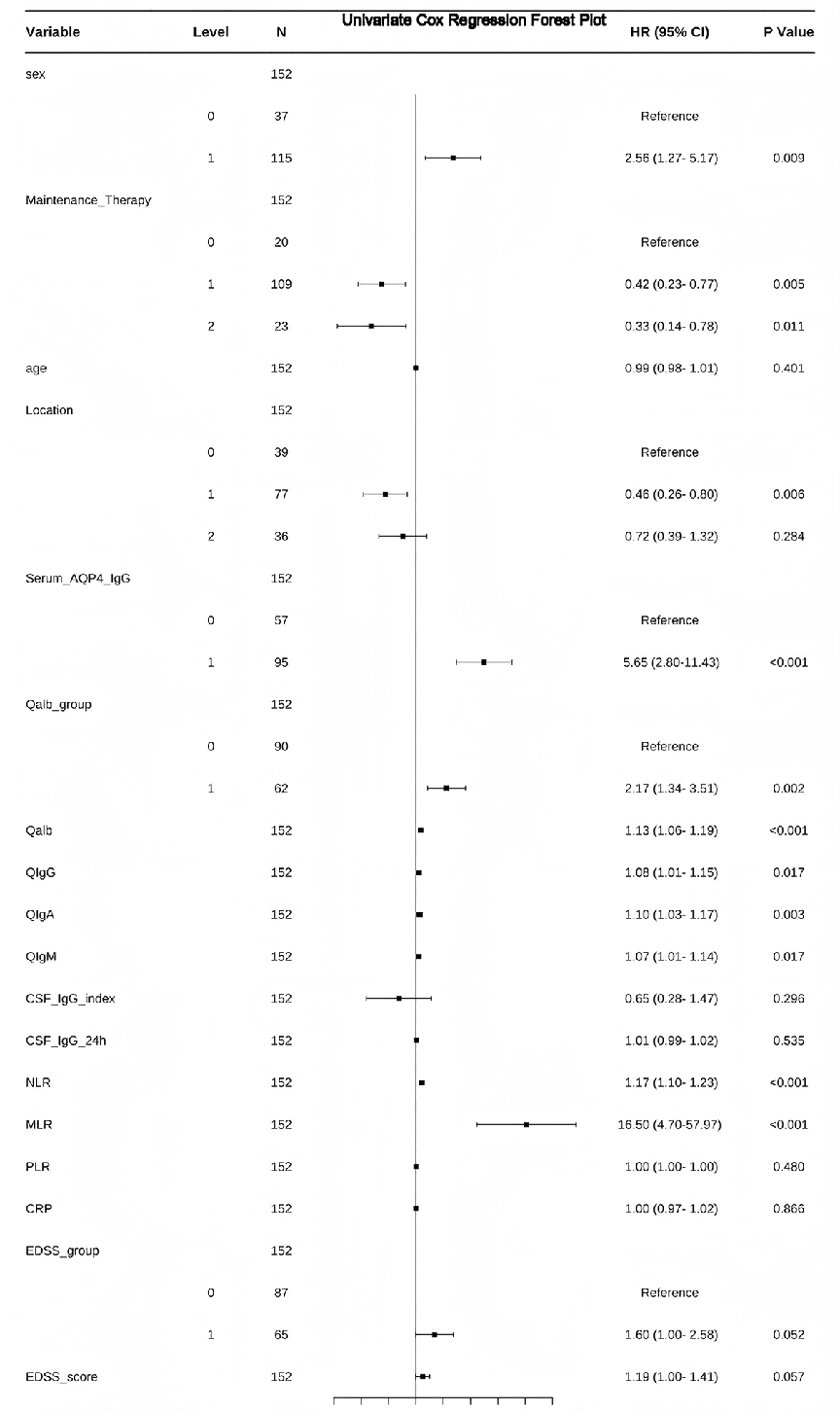


*Forest plot showing the results of univariable Cox proportional hazards regression analyses evaluating the association between baseline clinical characteristics, cerebrospinal fluid (CSF) indices, peripheral blood inflammatory markers, and maintenance therapy with time to first relapse in patients with neuromyelitis optica spectrum disorder (NMOSD).*

*Hazard ratios (HRs) are presented with corresponding 95% confidence intervals (CIs). Variables with HRs >1 indicate an increased risk of relapse, whereas HRs <1 indicate a protective effect.*

*Baseline factors assessed include sex, lesion location, serum aquaporin-4 immunoglobulin G (AQP4-IgG) serostatus, Expanded Disability Status Scale (EDSS) score, cerebrospinal fluid/serum albumin quotient (QAlb), cerebrospinal fluid immunoglobulin indices, neutrophil-to-lymphocyte ratio (NLR), monocyte-to-lymphocyte ratio (MLR), platelet-to-lymphocyte ratio (PLR), C-reactive protein (CRP), and maintenance immunotherapy category.*

*Variables reaching statistical significance (p < 0.05) in univariable analysis were subsequently entered into multivariable Cox regression models.*
