# Supplementary material for: Integrated Blood Inflammatory Ratios and Cerebrospinal Fluid Blood‒Brain Barrier Dysfunction Predict Relapse Risk in Neuromyelitis Optica Spectrum Disorder
Source: Brain Behav. 2026 Jun 12;16(6):e71481. doi: 10.1002/brb3.71481 (PMC13263635; doi:10.1002/brb3.71481)
Supplement: Supplementary file 4 — Figure S4. Kaplan–Meier curves of relapse‐free survival in patients with AQP4‐IgG positive vs. negative status. [file BRB3-16-e71481-s007.docx]

**Figure S4：Kaplan-Meier curves of relapse-free survival in patients with AQP4-IgG positive vs negative status**


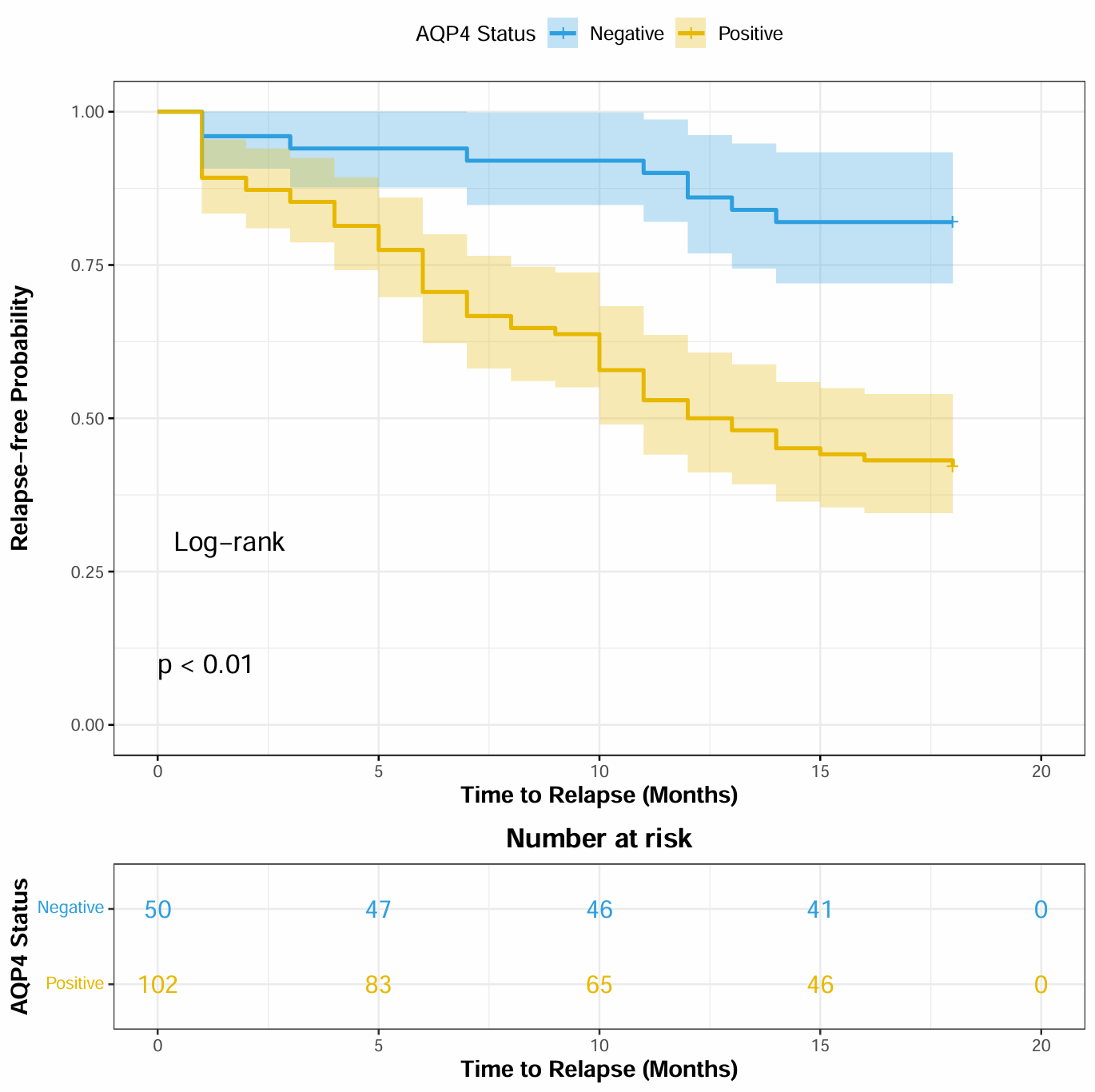


*The x-axis represents time to relapse (in months), and the y-axis represents the probability of remaining relapse-free. The blue curve indicates patients with AQP4-IgG negative status, and the yellow curve indicates patients with AQP4-IgG positive status. The Log-rank test was used to compare the difference between the two groups (p < 0.01). The “Number at risk” table below the plot shows the number of patients at risk in each group at the indicated time points (0, 5, 10, 15, and 20 months).*
