# Supplementary material for: Integrated Blood Inflammatory Ratios and Cerebrospinal Fluid Blood‒Brain Barrier Dysfunction Predict Relapse Risk in Neuromyelitis Optica Spectrum Disorder
Source: Brain Behav. 2026 Jun 12;16(6):e71481. doi: 10.1002/brb3.71481 (PMC13263635; doi:10.1002/brb3.71481)
Supplement: Supplementary file 5 — Figure S5. Subgroup multivariable Cox regression analysis in AQP4‐IgG seropositive patients. [file BRB3-16-e71481-s003.docx]

**Figure S5. Subgroup multivariable Cox regression analysis in AQP4-IgG seropositive patients.**


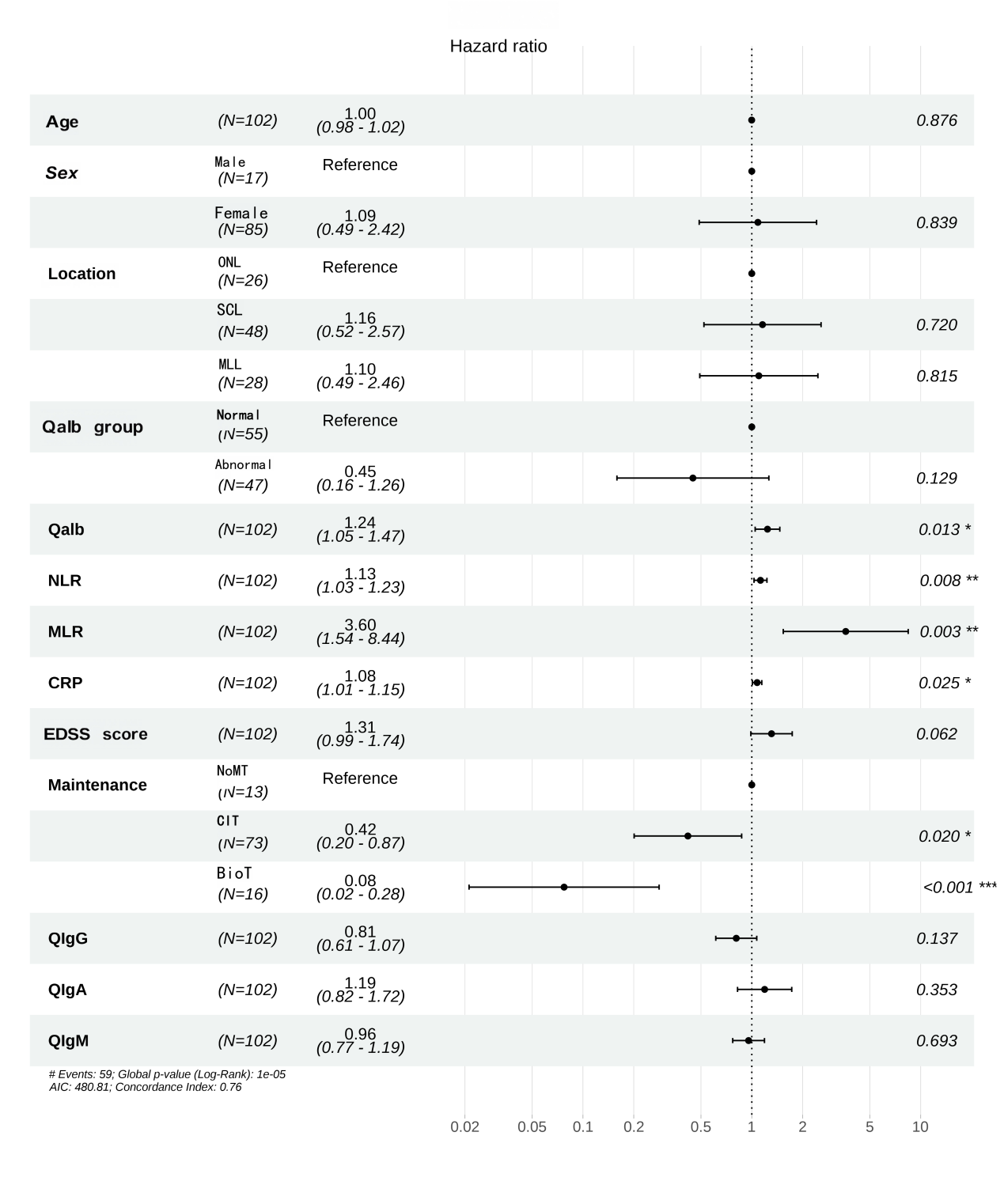

*Forest plot illustrating hazard ratios (HRs) and 95% confidence intervals (CIs) of predictors for relapse in AQP4-IgG seropositive patients. The direction and magnitude of effect estimates were consistent with those observed in the overall cohort, indicating the robustness of the model in clinically relevant populations. Horizontal lines denote 95% CIs, and the vertical dashed line represents HR = 1.0.*
