# Supplementary material for: Integrated Blood Inflammatory Ratios and Cerebrospinal Fluid Blood‒Brain Barrier Dysfunction Predict Relapse Risk in Neuromyelitis Optica Spectrum Disorder
Source: Brain Behav. 2026 Jun 12;16(6):e71481. doi: 10.1002/brb3.71481 (PMC13263635; doi:10.1002/brb3.71481)
Supplement: Supplementary file 6 — Figure S6. Performance and clinical utility of the prognostic model for relapse risk in NMOSD. [file BRB3-16-e71481-s005.docx]

**Figure S6. Performance and clinical utility of the prognostic model for relapse risk in NMOSD.**


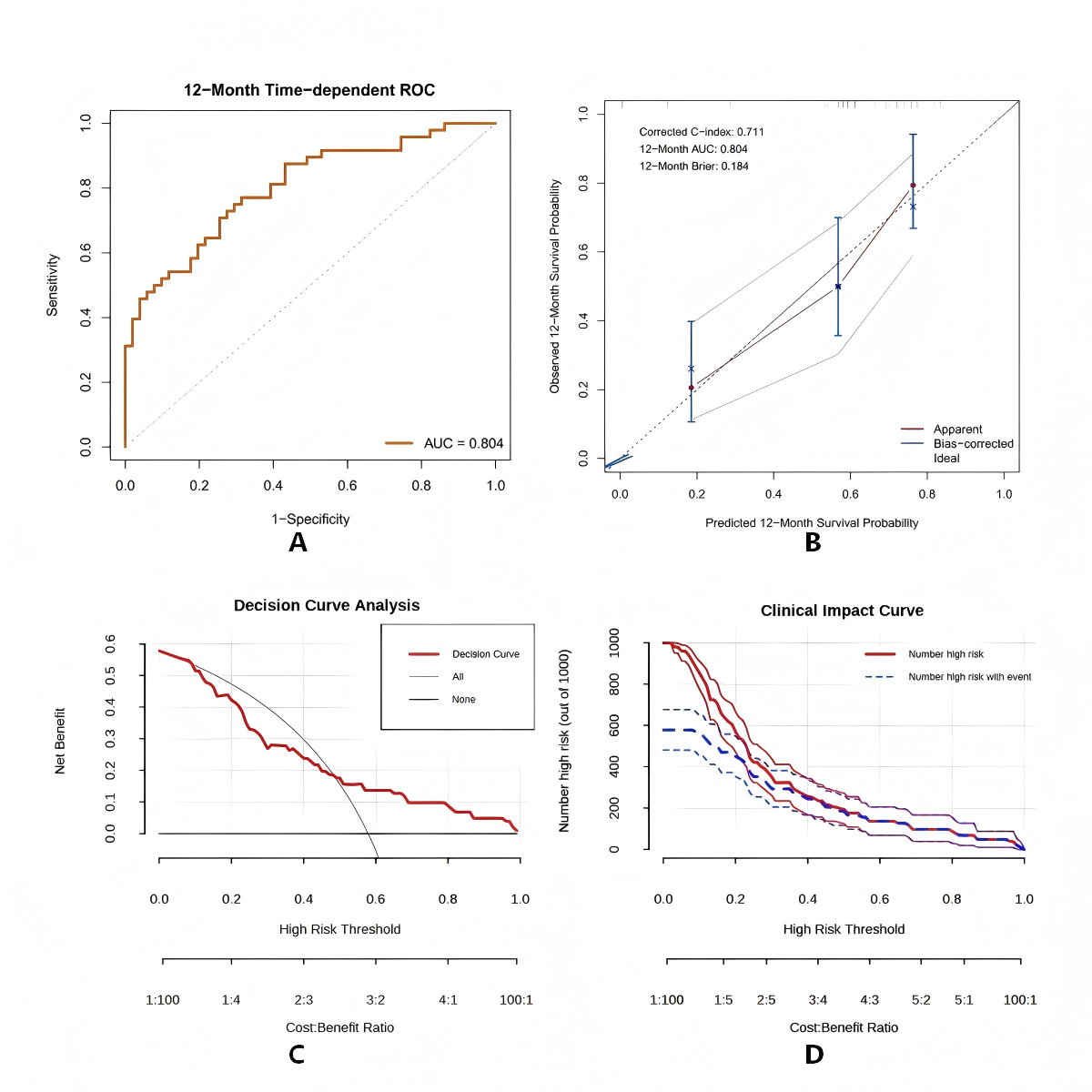

*(A) Time-dependent receiver operating characteristic (ROC) curve at 12 months, showing good discriminative ability with an area under the curve (AUC) of 0.804.
(B) Calibration plot for 12-month relapse prediction, demonstrating good agreement between predicted and observed probabilities. The optimism-corrected C-index was 0.711 and the Brier score was 0.184, indicating acceptable model calibration and overall performance.
(C) Decision curve analysis (DCA) showing that the model provides a higher net clinical benefit than the treat-all or treat-none strategies across a wide range of threshold probabilities.
(D) Clinical impact curve illustrating the estimated number of patients classified as high risk and the corresponding number of true relapse events across different threshold probabilities, supporting the potential clinical applicability of the model.*
