# Supplementary material for: Integrated Blood Inflammatory Ratios and Cerebrospinal Fluid Blood‒Brain Barrier Dysfunction Predict Relapse Risk in Neuromyelitis Optica Spectrum Disorder
Source: Brain Behav. 2026 Jun 12;16(6):e71481. doi: 10.1002/brb3.71481 (PMC13263635; doi:10.1002/brb3.71481)
Supplement: Supplementary file 7 — Figure S7. Hazard ratio for disease relapse by AQP4‐IgG titer. [file BRB3-16-e71481-s002.docx]

**Figure S7：Hazard ratio for disease relapse by AQP4-IgG titer**


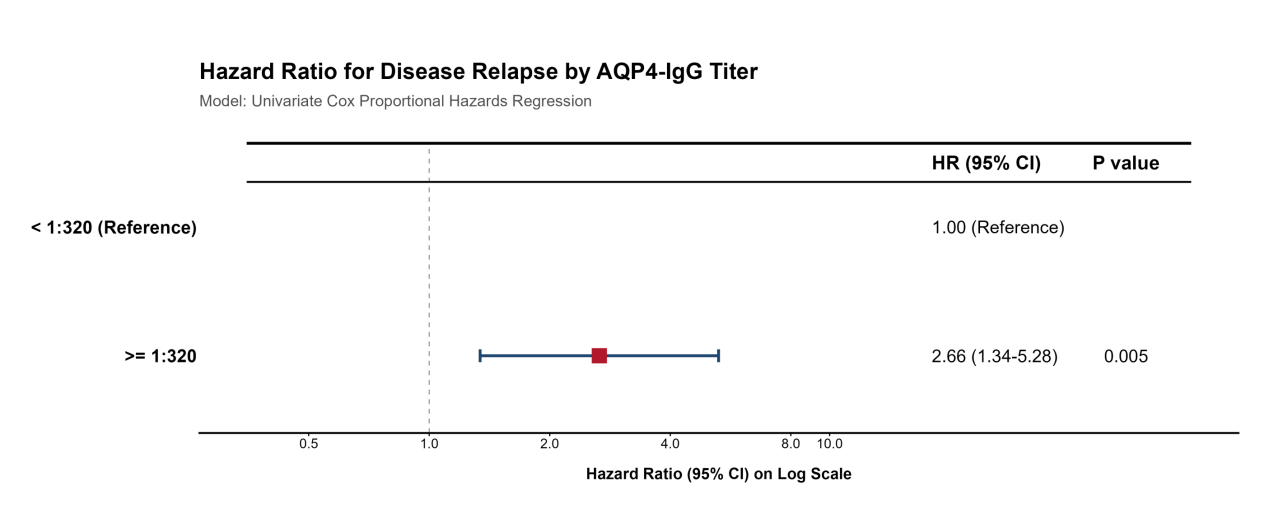


*This forest plot presents the results of a univariate Cox proportional hazards regression model evaluating the association between AQP4-IgG titer and disease relapse risk. The reference group is patients with an AQP4-IgG titer < 1:320 (HR = 1.00). Patients with a titer ≥ 1:320 show a hazard ratio of 2.66 (95% CI: 1.34–5.28, P = 0.005), indicating a significantly increased risk of relapse. The horizontal axis displays hazard ratios on a logarithmic scale; the red square represents the point estimate of the HR, and the horizontal blue line represents the 95% confidence interval.*
