# Supplementary material for: Integrated Blood Inflammatory Ratios and Cerebrospinal Fluid Blood‒Brain Barrier Dysfunction Predict Relapse Risk in Neuromyelitis Optica Spectrum Disorder
Source: Brain Behav. 2026 Jun 12;16(6):e71481. doi: 10.1002/brb3.71481 (PMC13263635; doi:10.1002/brb3.71481)
Supplement: Supplementary file 8 — Figure S8. Subgroup hazard ratios for disease relapse: interaction analysis between AQP4 status and inflammatory biomarkers. [file BRB3-16-e71481-s006.docx]

**Figure S8：Subgroup hazard ratios for disease relapse: interaction analysis between AQP4 status and inflammatory biomarkers**


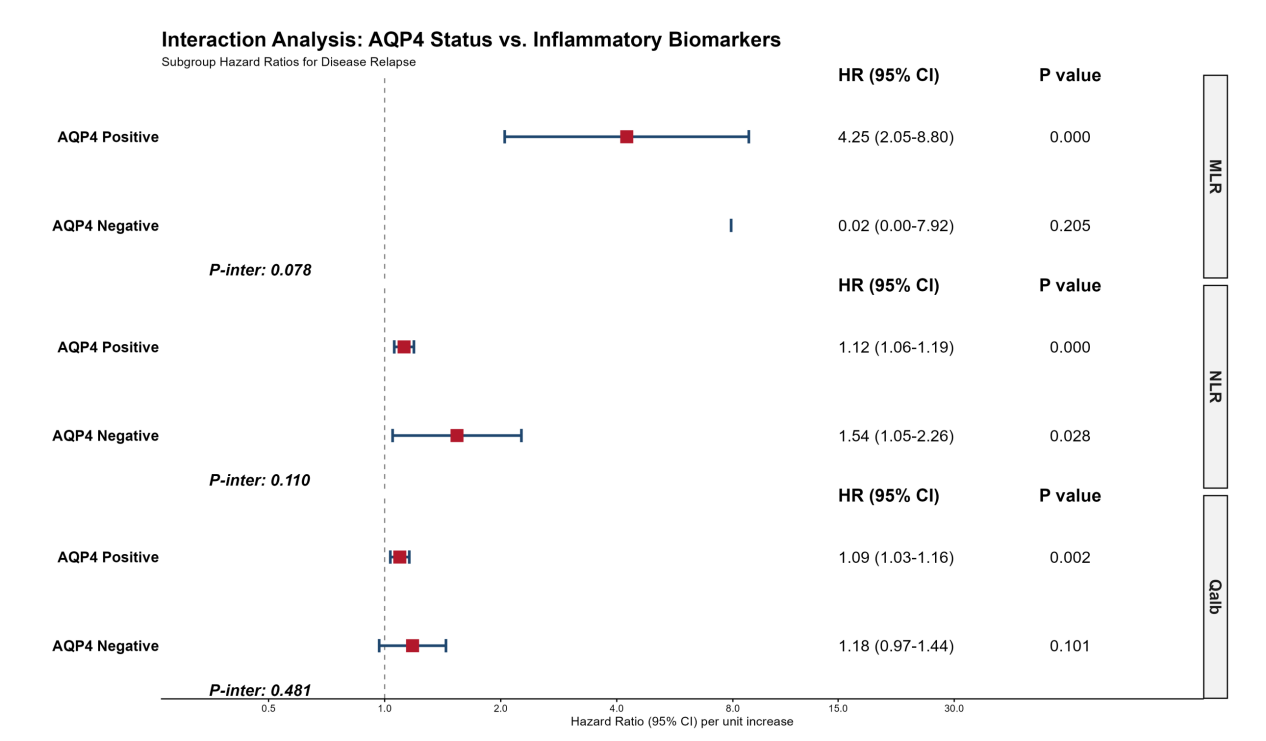
*This forest plot illustrates the interaction between AQP4-IgG status (positive vs. negative) and three inflammatory biomarkers (MLR, NLR, and Qalb) in relation to disease relapse risk, as estimated by Cox proportional hazards regression models.*

*Hazard ratios (HRs) are presented per unit increase in each biomarker, with 95% confidence intervals (CIs) shown as horizontal lines.The vertical dashed line at HR = 1.0 indicates the null effect.Red squares represent the point estimates of HRs for each subgroup.P-inter denotes the P-value for the interaction term between AQP4 status and the corresponding biomarker.Abbreviations: MLR,monocyte-to-lymphocyte ratio; NLR, neutrophil-to-lymphocyte ratio; Qalb, cerebrospinal fluid-to-serum albumin quotient.*
