# Supplementary material for: Integrated Blood Inflammatory Ratios and Cerebrospinal Fluid Blood‒Brain Barrier Dysfunction Predict Relapse Risk in Neuromyelitis Optica Spectrum Disorder
Source: Brain Behav. 2026 Jun 12;16(6):e71481. doi: 10.1002/brb3.71481 (PMC13263635; doi:10.1002/brb3.71481)
Supplement: Supplementary file 9 — Supplementary Table S1. Univariate Cox regression analysis of the association between AQP4‐IgG titer and relapse risk. [file BRB3-16-e71481-s001.docx]

**Supplementary Table S1：Univariate Cox regression analysis of the association between AQP4-IgG titer and relapse risk**

| Variables | Event | β | S.E | Z | P | HR (95%CI) |
| --- | --- | --- | --- | --- | --- | --- |
| AQP4-IgG Titer Group |  |  |  |  |  |  |
| Low Titer（n=21） | 11 |  |  |  |  | 1.00 (Reference) |
| High Titer（n=41） | 35 | 0.95 | 0.35 | 2.73 | 0.005 | 2.66 (1.34 ~ 5.28) |
| HR: Hazard Ratio, CI: Confidence Interval | | | | | | |

*Abbreviations: HR, hazard ratio; CI, confidence interval; β, regression coefficient; S.E., standard error; Z, Z-statistic; P, p-value.*
